# Establishing an innovative carbohydrate metabolic pathway for efficient production of 2-keto-l-gulonic acid in *Ketogulonicigenium robustum* initiated by intronic promoters

**DOI:** 10.1186/s12934-018-0932-9

**Published:** 2018-05-19

**Authors:** Cai-Yun Wang, Ye Li, Zi-Wei Gao, Li-Cheng Liu, Meng-Yue Zhang, Tian-Yuan Zhang, Chun-Fu Wu, Yi-Xuan Zhang

**Affiliations:** 1 School of Life Science and Biopharmaceutics, Shenyang Pharmaceutical University, 103 Wenhua Road, Shenyang, 110016 Liaoning People’s Republic of China; 2 Northeast Pharmaceutical Group Co., Ltd, Shenyang, 110026 People’s Republic of China; 3 Department of Biotechnology, School of Engineering, Nagoya University, Furo-cho, Chikusa-ku, Nagoya, 464-8603 Japan

**Keywords:** *Ketogulonicigenium*, Metabolic pathway, Genome analysis, Promoter, Phosphoketolase, Phosphotransacetylase, Acetyl-CoA

## Abstract

**Background:**

2-Keto-l-gulonic acid (2-KGA), the precursor of vitamin C, is currently produced by two-step fermentation. In the second step, l-sorbose is transformed into 2-KGA by the symbiosis system composed of *Ketogulonicigenium vulgare* and *Bacillus megaterium*. Due to the different nutrient requirements and the uncertain ratio of the two strains, the symbiosis system significantly limits strain improvement and fermentation optimization.

**Results:**

In this study, *Ketogulonicigenium robustum* SPU_B003 was reported for its capability to grow well independently and to produce more 2-KGA than that of *K. vulgare* in a mono-culture system. The complete genome of *K. robustum* SPU_B003 was sequenced, and the metabolic characteristics were analyzed. Compared to the four reported *K. vulgare* genomes, *K. robustum* SPU_B003 contained more tRNAs, rRNAs, NAD and NADP biosynthetic genes, as well as regulation- and cell signaling-related genes. Moreover, the amino acid biosynthesis pathways were more complete. Two species-specific internal promoters, P1 (*orf_01408* promoter) and P2 (*orf_02221* promoter), were predicted and validated by detecting their initiation activity. To efficiently produce 2-KGA with decreased CO_2_ release, an innovative acetyl-CoA biosynthetic pathway (XFP-PTA pathway) was introduced into *K. robustum* SPU_B003 by expressing heterologous phosphoketolase (*xfp*) and phosphotransacetylase (*pta*) initiated by internal promoters. After gene optimization, the recombinant strain *K. robustum*/pBBR-P1_*xfp2502*-P2_*pta2145* enhanced acetyl-CoA approximately 2.4-fold and increased 2-KGA production by 22.27% compared to the control strain *K. robustum*/pBBR1MCS-2. Accordingly, the transcriptional level of the 6-phosphogluconate dehydrogenase (*pgd*) and pyruvate dehydrogenase genes (*pdh*) decreased by 24.33 ± 6.67 and 8.67 ± 5.51%, respectively. The key genes responsible for 2-KGA biosynthesis, sorbose dehydrogenase gene (*sdh*) and sorbosone dehydrogenase gene (*sndh*), were up-regulated to different degrees in the recombinant strain.

**Conclusions:**

The genome-based functional analysis of *K. robustum* SPU_B003 provided a new understanding of the specific metabolic characteristics. The new XFP-PTA pathway was an efficient route to enhance acetyl-CoA levels and to therefore promote 2-KGA production.

**Electronic supplementary material:**

The online version of this article (10.1186/s12934-018-0932-9) contains supplementary material, which is available to authorized users.

## Background

**Fig. 1:**
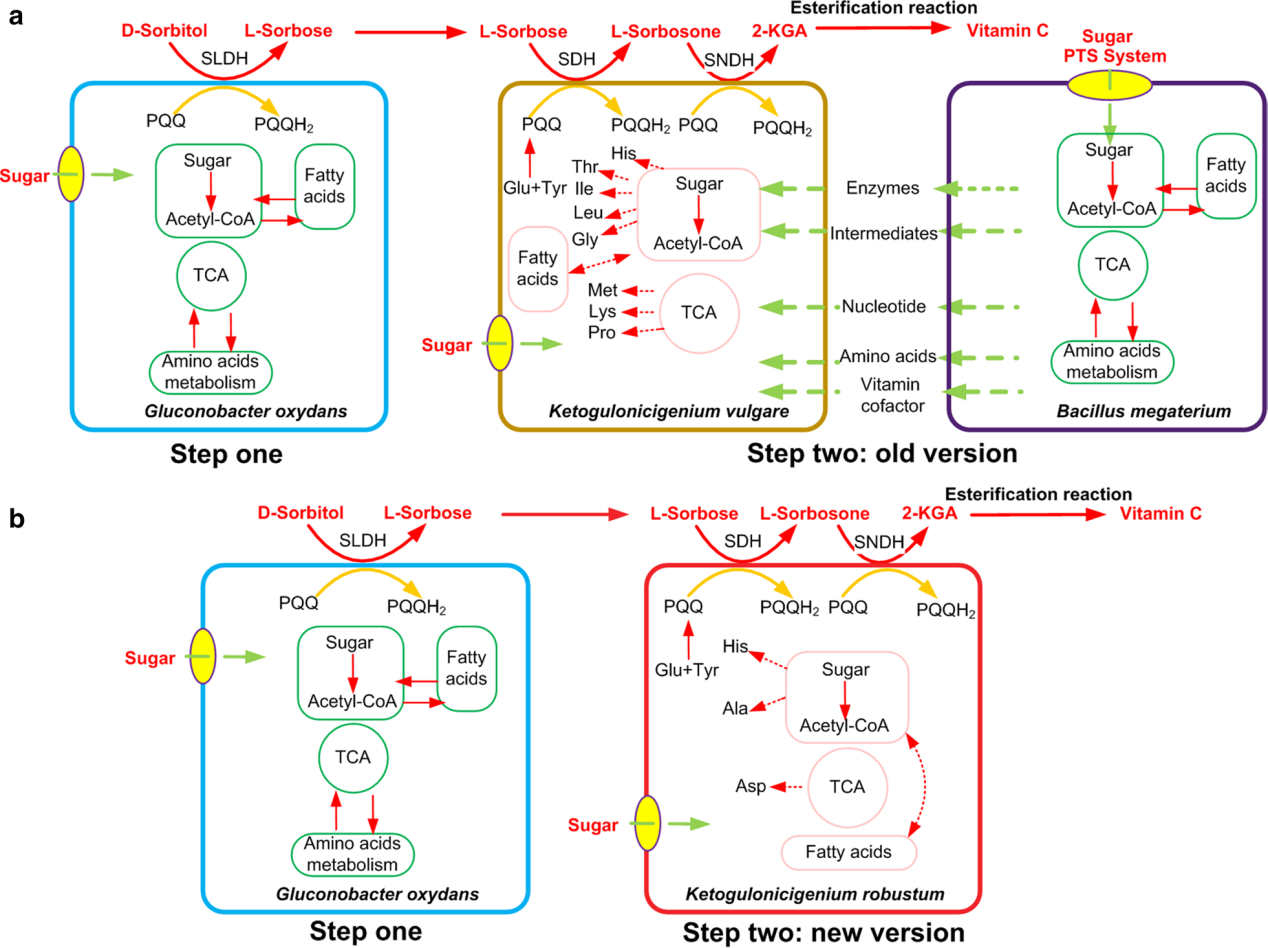
The alternatives of the two-step fermentation process of vitamin C. **a** The old version of the two-step fermentation process. **b** The new version of the two-step fermentation process

2-Keto-l-gulonic acid (2-KGA), the precursor of vitamin C, is currently produced by two-step fermentation from d-sorbitol. The first step is the generation of l-sorbose from d-sorbitol using *Gluconobacter oxydans*, and then l-sorbose is transformed into 2-KGA in the second step by the symbiosis system composed of *Ketogulonicigenium vulgare* and *Bacillus megaterium*. In the symbiosis system, *B. megaterium* is generally used as a companion strain that generates metabolites to assist the growth of *K. vulgare* and the accumulation of 2-KGA [1]. However, due to the different nutrient requirements and the uncertain optimal ratio of *K. vulgare* and *B. megaterium*, the co-culture system strongly limits the strain improvement and the fermentation optimization. Therefore, a mono-culture of *K. vulgare* is considered to be more cost-effective and manageable in the 2-KGA fermentation system. Nevertheless, the available industrial strain *K. vulgare* exhibited poor growth ability and lower 2-KGA productivity under mono-cultured condition [2]. Thus, to replace the co-culture fermentation system with one robust strain of *Ketogulonicigenium* could be revolutionary in the vitamin C industry. The alternatives for the two-step fermentation process are described in Fig. 1.

With the rapid development of high-throughput DNA sequencing technology, the whole genomes of several strains of *K. vulgare* have been completely sequenced and annotated in recent years [3–6], providing new perspectives to carry out detailed investigations on strain improvement. According to the genome annotation and metabolic network analysis, Liu et al. [7] found that *K. vulgare* were deficient in some key genes in the de novo biosynthesis of glycine, histidine, lysine, threonine, proline, isoleucine, methionine and leucine. Therefore, it was found that reconstruction of the biosynthetic pathways of threonine in *K. vulgare* Hkv604 increased 2-KGA production by 25.13%, and the fermentation period decreased by 28.57% when co-cultured with *Bacillus endophyticus* [8]. The defects in the metabolism of carbohydrates, amino acids and vitamins were the main reasons for the poor growth of *K. vulgare* Hbe602; however, the up-regulation of the tricarboxylic acid (TCA) cycle, as well as the metabolism of amino acids and nucleotide facilitated *K. vulgare* better growth [3].

Acetyl-CoA, as a central metabolic intermediate, plays important roles in cellular processes involved in the TCA cycle, lipid synthesis, amino acids metabolism and post-translational acetylation [9, 10]. Its main function is to deliver the acetyl group to the TCA cycle to be oxidized to CO_2_ and H_2_O, as well as energy in the form of ATP and GTP. The intermediates generated in the TCA cycle can be used as precursors for the biosynthesis of amino acids and lipids. In addition, the central position in metabolism endows acetyl-CoA with an important regulatory role. Acetyl-CoA serves as a substrate for the synthesis of lysine acetyltransferases (KATs), which transfer the acetyl group to lysines in histones and other proteins [11]. The acetylation of histones at the genes important for growth culminates in the expression of growth genes and a commitment to cell growth and division [9, 12]. To meet the cellular requirement, a variety of routes to synthesize acetyl-CoA have been formed, such as the decarboxylation of pyruvate, the catabolism of branched amino acids and the β-oxidation of fatty acids [13]. In the synthesis of acetyl-CoA from carbohydrate catabolism, the hexose is converted into d-glucose 6-phosphate (G6P) by phosphorylation and sequentially converted to d-ribulose 5-phosphate (Ru5P) by 6-phosphogluconate dehydrogenase (*pgd*) in the oxidative part of the pentose phosphate pathway (PPP). In the Embden–Meyerhof–Parnas (EMP) pathway, G6P is broken down to form pyruvate followed by the oxidative decarboxylation by pyruvate dehydrogenase (*pdh*). The byproduct of the two decarboxylation reactions is carbon dioxide, which is the main reason for the inefficiency of carbon utilization.

The phosphoketolase pathway has been described as an alternative carbon route due to the ability to bypass pyruvate decarboxylation [14] and has been successfully utilized to increase the relevant metabolites derived from acetyl-CoA in *Saccharomyces cerevisiae* [15–17], *Escherichia coli* [18] and *Methylomicrobium buryatense* [19]. The carbon flow towards acetyl-CoA in the phosphoketolase pathway is mainly driven by phosphoketolase and phosphotransacetylase. Phosphoketolase (XFP, EC 4.1.2.22) is a thiamine diphosphate-dependent (TPP) key enzyme for sugar metabolism in *Bifidobacterium*, and catalyzes the irreversible cleavage of d-fructose 6-phosphate (F6P) or/and d-xylulose 5-phosphate (X5P) to acetyl-phosphate (acetyl-P) and d-erythrose 4-phosphate (E4P) or d-glyceraldehyde 3-phosphate (G3P) [20]. The acetyl-P is subsequently converted to acetyl-CoA by phosphotransacetylase (PTA, EC 2.3.1.8) with complete carbon conservation [21]. Thereby, the phosphoketolase pathway can directly breakdown sugar phosphates into stoichiometric amounts of acetyl-CoA.

Recently, genetic engineering has been widely used in strain improvement in 2-KGA fermentation [8, 22, 23]. The promoter is a basic factor, playing a significant role in gene expression. The *E. coli*_*tufB* promoter, identified by An and Friesen [24], was successfully used in the conversion of d-sorbitol to 2-KGA in *G. oxydans_*G624 [25]. Shi et al. [26] isolated and identified the promoter *gHp*0169 from *G. oxydans* DSM 2003, which exhibited stronger activity than the *E. coli*_*tufB* promoter in the initiation of gene expression, and resulted in a twofold increase in the specific molar yield of 2-keto-d-gluconic acid. Besides the promoter of the sorbose dehydrogenase gene (Psdh) and the promoter of the sorbosone dehydrogenase gene (Psndh) in *K. vulgare* DSM 4025 [27], no other species-specific promoter in the *Ketogulonicigenium* sp. has been reported. Thereby, developing a series of well-characterized promoters is a necessary prerequisite to fine-tuning heterogeneous gene expression in *Ketogulonicigenium* sp.

In this study, a unique strain of *K. robustum* SPU_B003 with good growth ability and 2-KGA producing capacity in a mono-culture system, was reported. To fully understand the genome characteristics and to provide a comprehensive understanding of the genetic background for gene expression research, the whole genome of *K. robustum* SPU_B003 was sequenced. Based on the genome information, two putative promoters, P1 (*orf_01408* promoter) and P2 (*orf_02221* promoter), were predicted and identified by initiating the heterologous *gfp* gene expression in *K. robustum* SPU_B003. Compared to the promoter *E. coli*_*tufB*, the two promoters exhibited stronger activity, especially promoter P1, when initiating *gfp* expression.

To investigate the effect of oversupply of acetyl-CoA on cell growth and 2-KGA production, a higher efficiency acetyl-CoA biosynthetic pathway with lower carbon loss was constructed by heterologously expressing phosphoketolase (*xfp*) and phosphotransacetylase (*pta*) in *K. robustum* SPU_B003. The engineered strain *K. robustum*/pBBR-P1_*xfp2502*-P2_*pta2145* achieved the highest acetyl-CoA production, and reached a 22.27% increase in 2-KGA production. Accordingly, the transcriptional level of the genes *pgd* and *pdh* were decreased by 24.33 ± 6.67 and 8.67 ± 5.51%, respectively, and the transcriptional level of the genes sorbose dehydrogenase (*sdh*) and sorbosone dehydrogenase (*sndh*) were up-regulated at different levels. This may be because the XFP-PTA pathway is helpful in promoting the TCA cycle and generating α-ketoglutarate, the precursor of glutamate. Glutamate, as a building block, contributes to the biosynthesis of *pqq*A, the rate-determining step in pyrroloquinoline quinone (PQQ) biosynthesis. So, presumably, it is helpful to regulate electron transfer in the l-sorbose oxidation process and then affects the transcriptional levels of *sdh* and *sndh*.

## Methods

### Strains, plasmids and genes

All the strains and plasmids used in this work were described in Table 1. *K. robustum* SPU_B003 was isolated from a soil sample (E104°19′17.2″/N23°31′48.10″) and kept in our laboratory and in Microbial Resource Center of Shenyang Pharmaceutical University by cryopreservation and lyophilization. The *E. coli* WM3064 [28] was used as a donor strain in conjugation with *K. robustum* SPU_B003. The *Bifidobacterium animals* subsp. *lactis* was exchanged from the Bioresource Collection and Research Center (BCRC). The plasmid pBBR1MCS-2 [29] was used as an expression vector in *K. robustum* SPU_B003.

**Table 1:** Strains and plasmids used in this study

Names	Descriptions	Sources
Strains		
*E. coli* WM3064	RP4 (*tra*) in chromosome, DAP^−^	This lab
*B. animals* subsp. *lactis*	The strain contains *xfp* gene	BCRC
*E. coli* K12	The strain contains *pta* gene	This lab
*K. robustum* SPU_B003	2-KGA production strain	This lab
Plasmids		
pBBR1MCS-2	Broad host vector	This lab
pX551-*gfp*	Plasmid carries *gfp* gene	This lab
pBBR-P*tufB*_*gfp*	Recombinant plasmid carries *gfp* gene initiated by *E. coli*_*tufB* promoter	This study
pBBR-P1_*gfp*	Recombinant plasmid carries *gfp* gene initiated by P1 (*orf_01408*) promoter	This study
pBBR-P2_*gfp*	Recombinant plasmid carries *gfp* gene initiated by P2 (*orf_02221*) promoter	This study
pBBR-*xfp*-*pta*	Recombinant plasmid carries *xfp* and *pta* gene initiated by their original promoter, respectively	This study
pBBR-P1_*xfp*-P2_*pta*	Recombinant plasmid carries *xfp* and *pta* gene initiated by P1 and P2 promoter, respectively	This study
pBBR-P1_*xfp2502*-P2_*pta2145*	Recombinant plasmid carries optimized *xfp2502* and optimized *pta2145* gene initiated by P1 and P2 promoters, respectively	This study

### Genome sequence, annotation and metabolic pathway analysis

The complete genome sequence of *K. robustum* SPU_B003 was determined by the Chinese National Human Genome Center (Shanghai, China) using 454 single-end sequencing technology. A total of 123,540 reads with 20.49-fold coverage was generated. The reads were assembled into 29 large contigs (> 500 nucleotides) by using the 454 Newbler assembler (454 Life Sciences, Branford, CT). The relationship of contigs was determined by ContigScap [30], and the gaps between contigs were filled by PCR amplification followed by DNA sequencing.

The genome analysis was performed by Rapid Annotation using Subsystem Technology (RAST) [31]. The coding regions were distinguished by Gene Locator and Interpolated Markov ModelER (GLIMMER) [32]. The function annotation of open reading frames (ORFs) was carried out by using the Basic Local Alignment Search Tool (BLAST) [33] against the Kyoto Encyclopedia of Genes and Genomes (KEGG) database (Japan) [34]. Both tRNA and rRNA genes were identified by tRNAscan-SE [35] and RNAmmer [36], respectively. The NAD and NADP related genes were predicted by RAST. The metabolic networks were constructed using the KEGG database.

The nucleotide sequences immediately upstream of the ORF were subjected to BPROM [37] to predict promoters as well as the position of the − 10 and − 35 boxes.

### Genetic manipulation

The *gfp* gene was cloned from the pX551-*gfp* plasmid and was used as a reporter. The promoter *E. coli*_*tufB* was cloned from *E. coli* K12 by PCR according to the sequence reported by An and Friesen [24]. The fusion of the predicted promoter P1 sequence and the *gfp* fragment was made by overlap extension PCR, followed by insertion into the expression vector pBBR1MCS-2 to obtain the recombinant plasmid pBBR-P1_*gfp*. The same methods were used to obtain the plasmid pBBR-P2_*gfp* and pBBR-P*tufB*_*gfp*.

The gene *xfp* with its original upstream promoter were amplified from *B. animals*, and *pta* with its original promoter were from *E. coli* K12. The PCR fragments were digested with restriction enzyme and ligated into pBBR1MCS-2 to produce pBBR-*xfp*-*pta*. The original promoters of *xfp* and *pta* were replaced by promoter P1 and P2 of *K. robustum* SPU_B003, respectively, herein to construct the plasmid pBBR-P1_*xfp*-P2_*pta*. Due to the codon preference of *K. robustum* SPU_B003, the genetic bases of heterologous *xfp* and *pta* were optimized to *xfp2502* and *pta2145* and synthesized by Sangon Biotech (Shanghai Co., Ltd). The codon frequency table and the optimized parameters were listed in Additional file 1: Tables S1, S2. Thus, another plasmid pBBR-P1_*xfp2502*-P2_*pta2145* was built. Afterwards, the plasmids were transferred from *E. coli* WM3064 to *K. robustum* SPU_B003 by conjugation.

For the orthogonality test, the promoters P1 and P2 were assembled with *xfp2502* and *pta2145* (4 combinations) on pBBR1MCS-2. The orthogonality test was conducted by the same method described above. All the primers and DNA sequences used in this work were listed in Additional file 1: Tables S3, S4.

### Culture media and growth condition

*Escherichia coli* was cultured in Luria–Berta (LB) medium at 37 °C, and 25 μg/mL diaminopimelic acid (DAP) was added when *E. coli* WM3064 was cultivated. Seed medium for *K. robustum* SPU_B003 was composed of 20 g/L corn steep liquor, 10 g/L peptone, 10 g/L sorbitol, 10 g/L mannitol and 10 g/L CaCO_3_ (pH 6.5). Fermentation medium for *K. robustum* SPU_B003 contained 20 g/L corn steep liquor, 40 g/L l-sorbose, 1 g/L MgSO_4_, 0.04 g/L nicotinamide, 0.37 g/L calcium pantothenate, 0.168 g/L aminobenzoic acid, and 25 g/L CaCO_3_ (pH 7.0). An appropriate kanamycin quantity was added to the medium when needed.

### Fluorescence intensity determination

The recombinant *K. robustum* SPU_B003 was cultured in seed medium for 24 h, harvested at 6000 g for 10 min, and washed twice with PBS buffer. The whole cell fluorescence (RFU/OD_600_, the relative fluorescence unit divided by the corresponding cell density) was determined according to Xu et al. [38] by a fluorescence microplate reader (Infinite M200 Pro, Tecan, Mannedorf, Zurich, Switzerland). The excitation and emission wavelength were 485 and 535 nm, respectively. The cell density was determined at 600 nm by a microplate reader. Meanwhile, the cells were photographed by using a fluorescence microscope (Olympus BX53F, Tokyo, Japan).

### Enzyme activity assays

The phosphoketolase activity was detected spectrophotometrically at 505 nm by the formation of ferric acetyl hydroxamate [39]. One unit of phosphoketolase activity was defined as the amount of protein forming 1 μmol of acetyl phosphate (acetyl-P) per 1 min from fructose 6-phosphate. Specific activity was defined as units per 1 mg of protein. Protein concentration was determined according to the method described by Bradford with bovine serum albumin as standard [40].

The phosphotransacetylase activity was measured by the CoA-dependent arsenolytic decomposition of acetyl-P according to the procedure of Suzuki [41]. One unit of phosphotransacetylase was defined as the amount of protein extract that catalyzed the decomposition of 1 μmol of acetyl-P per minute.

### Intracellular acetyl-CoA quantification

Intracellular acetyl-CoA was measured by using an Acetyl-CoA Assay Kit (Suzhou Comin Biotechnology Co., Ltd). Malate dehydrogenase (MDH) catalyzed malic acid and NAD to oxaloacetic acid and NADH. Then, citrate synthase (CS) catalyzed acetyl-CoA and oxaloacetic acid to form citric acid and CoA. Through the coupled reaction of MDH and CS, the content of acetyl-CoA was proportional to the formation rate of NADH. Therefore, the concentration of acetyl-CoA was determined by measuring the rate of absorbance rise at 340 nm. The sample was normalized by protein concentration [40].

### Quantitative real time PCR

The cells were collected at 24 h of fermentation by centrifugation at 10,000*g* for 10 min at 4 °C. The total RNA was isolated by using the TriZol reagent (Vazyme Biotech Co., Ltd), and then extracted with chloroform. The RNA was precipitated with isopropanol and washed with 75% ethanol. After dissolving in RNase-free water, the sample concentration was quantified by a NanoDrop 2000 spectrophotometer (Thermo Fisher Scientific, USA). Quantitative real time PCR was conducted with GoTaq qPCR Master Mix [Promega (Beijing) Biotech Co., Ltd] on Stratagene Mx3000P (Agilent Technologies, Inc, USA). The 16S rRNA gene was used as an internal standard. The primers and DNA sequences were listed in Additional file 1: Tables S3, S4.

### Cell density determination

The cell density was measured spectrophotometrically at 600 nm after dissolving CaCO_3_ with 100 mM HCl.

### 2-KGA concentration determination

The concentration of 2-KGA in fermentation broth was measured by the high-performance liquid chromatography (HPLC) using an amino column. The mobile phase used was acetonitrile-KH_2_PO_4_ (5%/95%, v/v) with a flow rate of 0.6 mL/min.

## Results and discussion

### General genomic properties of *K. robustum* SPU_B003

In this study, a robust strain, *K. robustum* SPU_B003, was reported, and its growth characteristics, utilization of resources and enzyme activities were listed in Additional file 1: Table S5. Phylogenetic analysis based on 16S rRNA, revealed that *K. robustum* SPU_B003 is classified in the same clade with *K. robustum* X6L [42] (Additional file 1: Figure S1). The genome consisting of one circular chromosome and five circular plasmids, was deciphered and deposited in the GenBank database with the accession numbers CP019937 (chromosome, 2,452,467 bp, GC content 62.3%), CP019938 (plasmid 1, 226,430 bp, GC content 63.5%), CP019939 (plasmid 2, 19,694 bp, GC content 54.6%), CP019940 (plasmid 3, 7028 bp, GC content 62.5%), CP019941 (plasmid 4, 4005 bp, GC content 53.0%) and CP019942 (plasmid 5, 4211 bp, GC content 61.8%). The chromosome encodes 2316 proteins, 61 tRNAs and 18 rRNAs, while the five plasmids, plasmids 1–5, encode 197, 16, 8, 3 and 5 proteins, respectively. SDH and SNDH are the key enzymes responsible for the conversion of l-sorbose to 2-KGA [43, 44]. Accordingly, three *sdh* genes (*orf_02251* of *sdh*-1, *orf_02271* of *sdh*-2, *orf_p00164* of *sdh*-3) and two *sndh* genes *(orf_00349* of *sndh*-1, *orf_01127* of *sndh*-2) were found in the genome of *K. robustum* SPU_B003. The *sdh*-3 gene is located in plasmid 1, and the other genes are located in the chromosome.

**Fig. 2:**
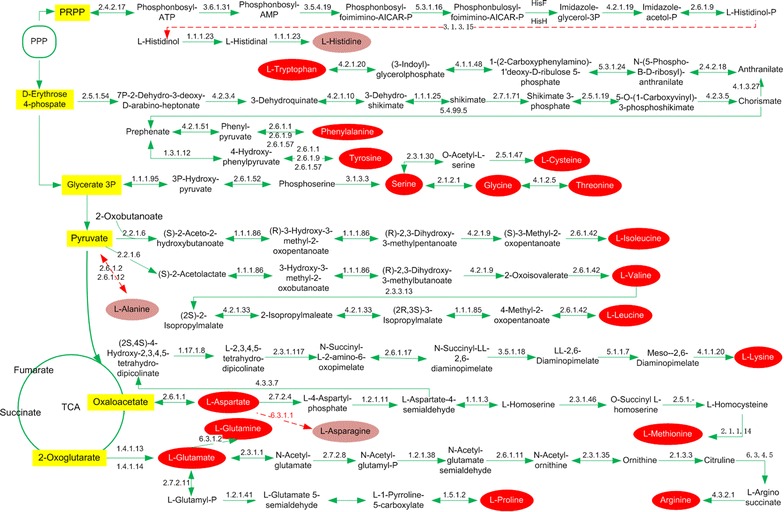
Reconstruction of amino acid biosynthesis pathway based on KEGG database. The absent genes are marked with dotted lines. The dark red indicates that the amino acid biosynthesis pathways are incomplete

Compared with the published genome, species-specific characteristics of *K. robustum* SPU_B003 were presented in Table 2. The numbers of tRNAs (61) and rRNA (18) in *K. robustum* SPU_B003 are more than those in the four reported *K. vulgare*. In the dividing cell, the amount of tRNAs determines codon translation efficiency [45]. rRNAs are the key players in the ribosome which carries out protein synthesis. Therefore, the abundance of tRNAs and rRNAs can contribute to the biosynthesis of protein in *K. robustum* SPU_B003. The number of NAD and NADP biosynthetic genes (10) is more than *K. vulgare* WSH-001 (8), SKV (8) and Hbe602 (8). *K. robustum* SPU_B003 has abundant regulation and cell signaling system related genes (32) in response to changes in their external environment. In addition, unlike *K. vulgare* WSH-001 [7], *K. robustum* SPU_B003 has a more complete amino acids biosynthetic pathway except for histidine, alanine and asparagine (Fig. 2). All these factors probably facilitated the ability of *K. robustum* SPU_B003 to grow independently.

**Table 2:** The genome features of several strains of *Ketogulonicigenium* sp.

	*K. robustum* SPU_B003	*K. vulgare* WSH-001	*K. vulgare* Y25	*K. vulgare* SKV	*K. vulgare* Hbe602
Chromosome (Mb)	2.45	2.77	2.78	2.76	2.77
Plasmids	5	2	2	1	2
tRNA	61	56	59	58	58
rRNA	18	15	15	15	15
NAD and NADP	10	8	10	8	8
Regulation and cell signaling	32	25	27	25	25
Protein	2545	3054	2905	2851	3178
Gene	2674	3198	3286	3067	3281
SDH	3	5	5	6	6
SNDH	2	2	2	1	2
Reference	This work	[5]	[6]	[4]	[3]
Accession number	CP019937 CP019938 CP019939 CP019940 CP019941 CP019942	CP002018 CP002019 CP002020	CP002224 CP002225 CP002226	CP016592 CP016593	CP012908 CP012909 CP012910

**Fig. 3:**
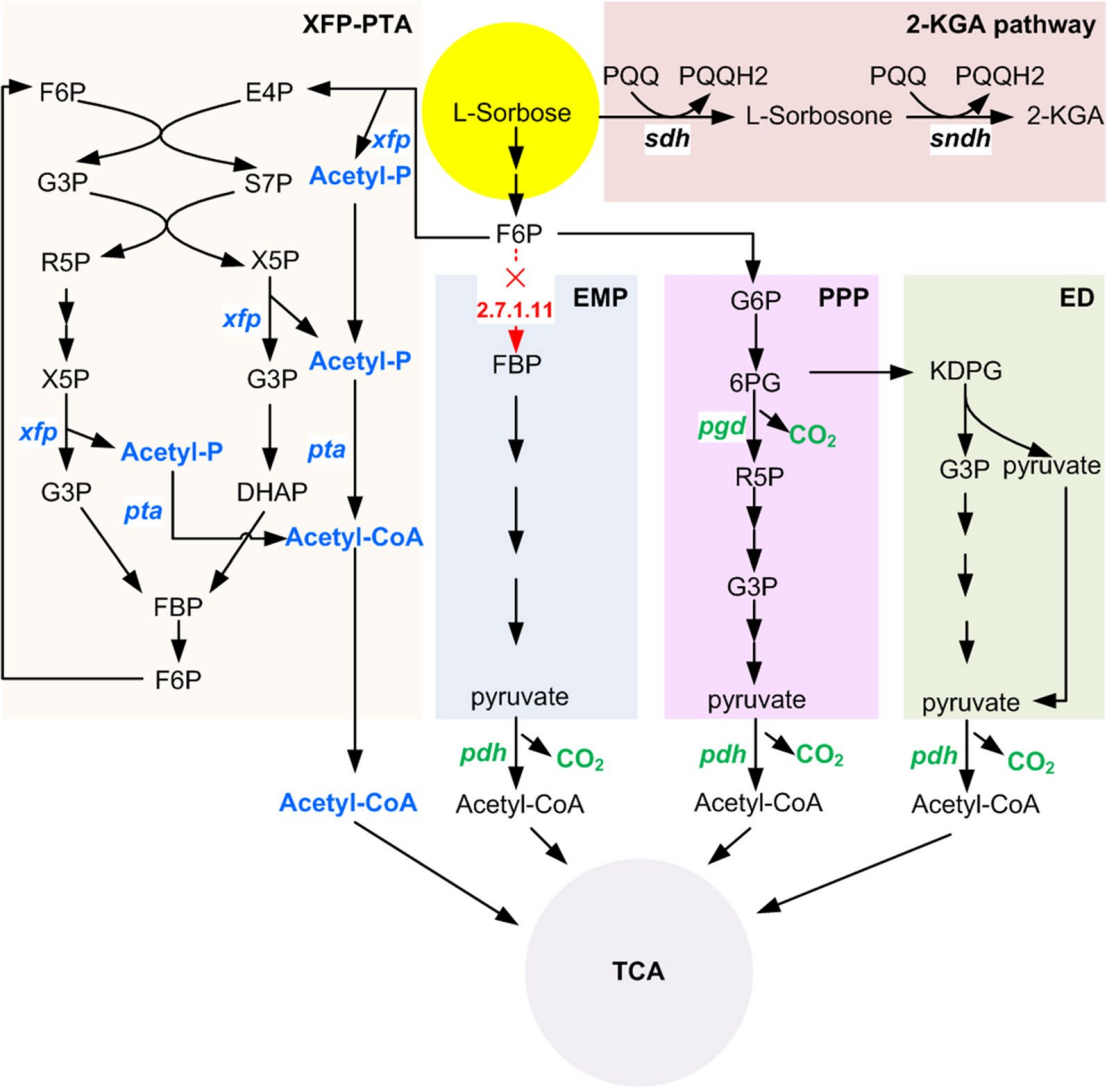
Metabolic pathway of *K. robustum* SPU_B003. Left panel, the new XFP-PTA pathway; lower panel, the EMP, PPP, ED and TCA pathways; upper panel, 2-KGA pathway. The metabolites and the genes catalyzed by *xfp/xfp2502* and *pta/pta2145* are indicated by the bolded blue font. The absent gene (EC 2.7.1.11) is indicated by the dotted red arrow. The CO_2_ release reactions are indicated in bold green. Metabolite abbreviations: fructose 1,6-bisphosphate (FBP), 6-phosphogluconate (6PG), 2-keto-3-deoxy-6 phosphogluconic acid (KDPG), dihydroxyacetone phosphate (DHAP)

According to the gene annotation and metabolic network analysis, carbohydrate metabolism pathways were studied, showing the presence of the genes encoding all the enzymes of PPP, Entner–Doudoroff (ED) pathway and TCA cycle, and the deficiency of the gene encoding 6-phosphofructokinase (EC 2.7.1.11) (Fig. 3). Therefore, the PPP pathway and ED pathway were the main central carbohydrate metabolism routes to link up with the TCA cycle. Considering the low efficiency (2/9 carbon loss) in the synthesis of acetyl-CoA in the conventional pathway, a new acetyl-CoA biosynthetic pathway was established by expressing heterologous phosphoketolase (*xfp*) and phosphotransacetylase (*pta*) in *K. robustum* SPU_B003 (Fig. 3). In the new pathway, l-sorbose was converted to F6P after a series of reactions, and F6P was directly broken down to acetyl-P and E4P by phosphoketolase (XFP). Then, E4P molecules regenerated F6P by carbon rearrangement. After the reactions catalyzed by transketolase and transaldolase, F6P and E4P were broken down to X5P and d-ribose 5-phosphate (R5P). X5P was broken down to acetyl-P and G3P. Finally, the acetyl group was transferred to coenzyme A catalyzed by phosphotransacetylase (PTA) to produce acetyl-CoA. Therefore, theoretically, 1 mol of l-sorbose can produce 3 mol of acetyl-CoA without CO_2_ production, which greatly improves the utilization rate of l-sorbose and the content of intracellular acetyl-CoA. The improvement of acetyl-CoA content helps to promote the TCA cycle, and then generates various intermediates for amino acid biosynthesis and energy to meet biological requirements. In addition, acetyl-CoA as a signal initiates the cellular growth program by promoting the acetylation of histones specifically at growth genes.

### Promoter analysis

The upstream sequences of *orf_01408* and *orf_02221*, which encode 50 s ribosomal protein L13 and DNA-directed RNA polymerase beta subunit, were predicted as promoter P1 and P2 by BPROM, separately. The spacing between the putative − 10 and − 35 regions were 13 bp for P1 and 15 bp for P2, which met the general pattern of most bacterial promoters.

### Promoter activity identification

**Fig. 4:**
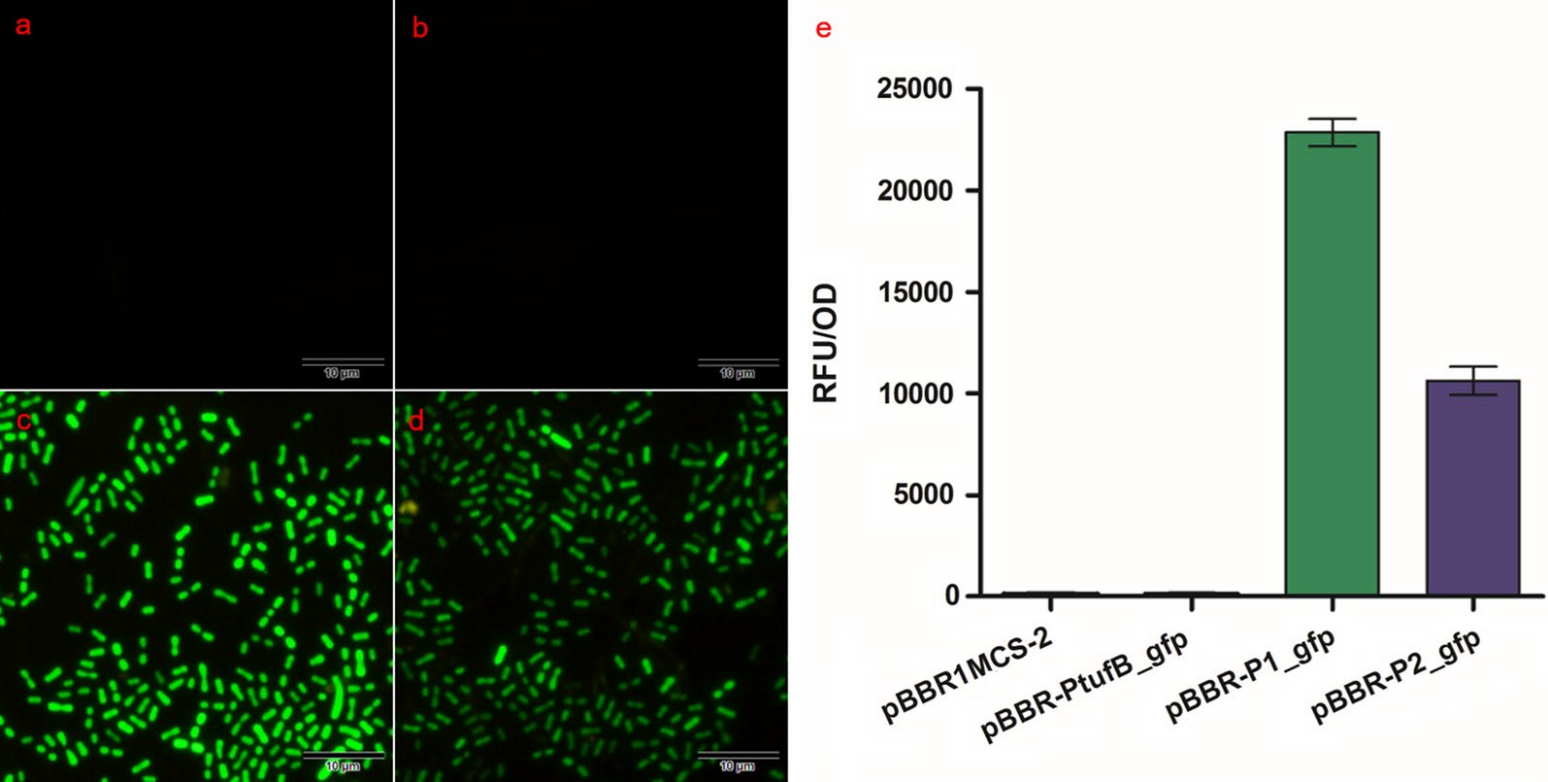
Detection of intronic promoter activity. **a**–**d** The images of *gfp* gene expression in recombinant strains initiated by different promoters. Strains harboring plasmids of (**a**) pBBR1MCS-2, (**b**) pBBR-P*tufB*_*gfp* (**c**) pBBR-P1_*gfp* or (**d**) pBBR-P2_*gfp* were photographed with a fluorescence microscope. **e** The whole cell relative fluorescence intensity (RFU/OD) of different strains was measured using a fluorescence microplate reader. Data in **e** represents the mean ± SD of 3 replicates

The activities of promoter P1 and P2 were compared with *E. coli*_*tufB*, a commonly used promoter, by measuring the *gfp* fluorescence intensity (RFU/OD) in *K. robustum* SPU_B003. As shown in Fig. 4, the recombinant strains *K. robustum*/pBBR-P1_*gfp* (RFU/OD 22,187 ± 664.6) and *K. robustum*/pBBR-P2_*gfp* (RFU/OD 10,617 ± 697.8) exhibited prominent fluorescence, while the signals of control *K. robustum*/pBBR1MCS-2 (RFU/OD 52.81 ± 36.37) and *K. robustum*/pBBR-P*tufB*_*gfp* (RFU/OD 121.4 ± 17.79) are minimal. Moreover, the intensity of promoter P1 was stronger than promoter P2. The results significantly indicated that both of the promoters were functional in initiating heterologous gene expression in *K. robustum* SPU_B003.

### Enzymatic activity assay

To find the most effective combination of promoters and heterologous genes, the orthogonality test was conducted on pBBR1MCS-2. Results showed that P1 for *xfp/xfp2502* and P2 for *pta/pta2145* displayed the most effective performance (Additional file 1: Figure S2).

**Fig. 5:**
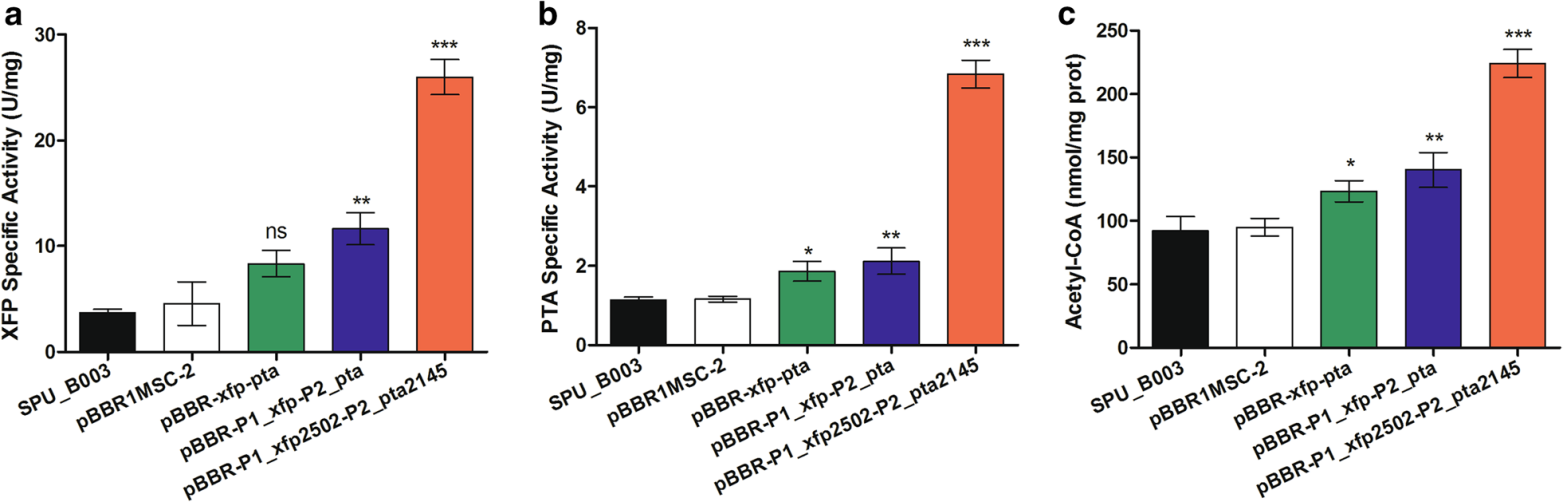
Expression of the heterologous genes *xfp/xfp2502* and *pta/pta2145* in *K. robustum* SPU_B003. **a**, **b** Enzymatic activity of *xfp/xfp2502* and *pta/pta2145* in different strains. **c** Acetyl-CoA level in vivo. Comparison was performed with control strain *K. robustum*/pBBR1MCS-2. Data represent the mean ± SD of 3 replicates. *, ** and *** Significant difference at *p* < 0.05, 0.01 and 0.001, respectively

As shown in Fig. 5a, the specific activity of XFP in *K. robustum*/pBBR-P1_*xfp2502*-P2_*pta2145* (26.00 ± 1.67 U/mg) and *K. robustum*/pBBR-P1_*xfp*-P2_*pta* (11.63 ± 1.52 U/mg) exhibited significant improvement compared to control *K. robustum*/pBBR1MCS-2 (4.56 ± 2.06 U/mg), with the former having a higher activity than the latter, indicating that the optimized gene *xfp2502* can be efficiently translated into protein in *K. robustum* SPU_B003. The XFP activity in *K. robustum*/pBBR-P1_*xfp*-P2_*pta* (11.63 ± 1.52 U/mg) was higher than that in *K. robustum*/pBBR-*xfp*-*pta* (8.24 ± 1.24 U/mg), which demonstrated that promoter P1 was stronger than the original promoter of *xfp*.

The specific activity of PTA exhibited a similar trend to XFP (Fig. 5b). Strain *K. robustum*/pBBR-P1_*xfp2502*-P2_*pta2145* (6.83 ± 0.35 U/mg) exhibited the best performance in the three engineered strains, which illustrated that the optimized gene *pta2145* was expressed in *K. robustum* SPU_B003 with high efficiency and that promoter P2 was stronger than the original promoter of *pta*. Therefore, the foreign genes could well exhibit enzymatic activity initiated by intronic promoters in *K. robustum* SPU_B003.

### Intracellular acetyl-CoA quantification

To verify the introduced XFP-PTA pathway was functional, the level of its downstream product, acetyl-CoA, was determined by the coupled reaction of MDH and CS, in which the content of acetyl-CoA was proportional to the formation rate of NADH. As shown in Fig. 5c, the acetyl-CoA level in strain *K. robustum*/pBBR-P1_*xfp2502*-P2_*pta2145* (224.10 ± 11.14 nmol/mg prot) increased by approximately 2.4-folds compared to *K. robustum*/pBBR1MCS-2 (94.88 ± 7.00 nmol/mg prot) and also higher than strain *K. robustum*/pBBR-*xfp*-*pta* (123.20 ± 8.44 nmol/mg prot) and strain *K. robustum*/pBBR-P1_*xfp*-P2_*pta* (140.10 ± 13.64 nmol/mg prot). This indicated that the introduced *xfp/xfp2502* and *pta/pta2145* were functional in the biosynthetic process of acetyl-CoA and that the optimized genes, *xfp2502* and *pta2145,* could further increase intracellular acetyl-CoA levels. The carbon flow through the new XFP-PTA pathway was successfully constructed in *K. robustum* SPU_B003.

### 2-KGA production assay

**Fig. 6:**
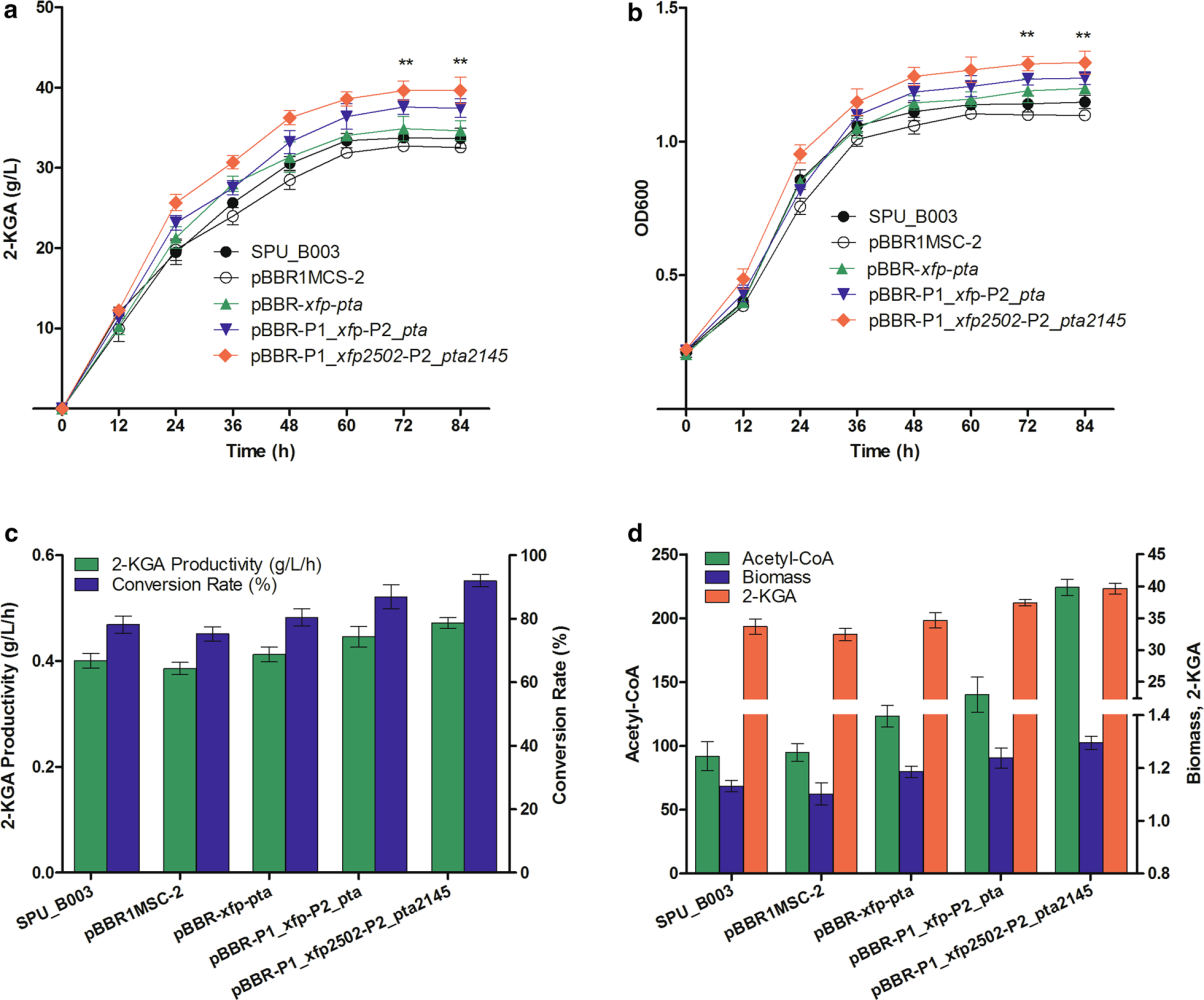
Effect of established XFP-PTA pathway on 2-KGA production. **a** Time course of 2-KGA production in the fermentation. **b** The growth curve of different strains. **c** 2-KGA productivity and l-sorbose conversion rate. **d** The relationship of acetyl-CoA, biomass and 2-KGA in fermentation. Comparison was performed with control strain *K. robustum*/pBBR1MCS-2. Data represent the mean ± SD of 3 replicates. *, ** and *** Significant difference at *p* < 0.05, 0.01 and 0.001, respectively

To assess the effect of the new metabolic pathway on cell growth and 2-KGA production, all strains were cultivated in fermentation medium containing 40 g/L l-sorbose in flasks. The concentration of 2-KGA in fermentation broth was measured by HPLC using acetonitrile-KH_2_PO_4_ (5%/95%, v/v) as mobile phase with a flow rate of 0.6 mL/min. The production of 2-KGA and biomass were described in Fig. 6a, b. The 2-KGA production of wild type strain *K. robustum* SPU_B003 reached 33.68 ± 2.17 g/L with a conversion rate of 78.13 ± 5.03% and a biomass OD_600_ of 1.15 ± 0.043 at 84 h. However, the control strain *K. robustum*/pBBR1MCS-2 exhibited a slight decrease in 2-KGA yield (32.42 ± 0.96 g/L, conversion rate 75.21 ± 2.23%) and in biomass (OD_600_ 1.10 ± 0.042) at the end of the fermentation. These indicated that the metabolic burden caused by the blank plasmid had little inhibitory effect on cell growth and 2-KGA production.

After introducing the heterologous genes *xfp/xfp2502* and *pta/pta2145*, the 2-KGA production of *K. robustum*/pBBR-*xfp*-*pta* and *K. robustum*/pBBR-P1_*xfp*-P2_*pta* were 34.63 ± 2.17 g/L (conversion rate 80.34 ± 5.03%) and 37.44 ± 2.01 g/L (conversion rate 86.86 ± 4.66%), respectively, which was increased by 6.82 and 15.48%, respectively, compared to control strain *K. robustum*/pBBR1MCS-2 (32.42 ± 0.96 g/L, conversion rate 75.21 ± 2.23%). The biomass of *K. robustum*/pBBR-*xfp*-*pta* (OD_600_ 1.20 ± 0.025) and *K. robustum*/pBBR-P1_*xfp*-P2_*pta* (OD_600_ 1.24 ± 0.041) also increased at different levels. Strain *K. robustum*/pBBR-P1_*xfp2502*-P2_*pta2145* exhibited the highest 2-KGA yield (39.64 ± 2.84 g/L, conversion rate 91.96 ± 6.59%) and biomass (OD_600_ 1.30 ± 0.075) (Fig. 6a, b), which increased by 22.27% in 2-KGA production and 16.75% in 2-KGA conversion rate in contrast to control strain. Moreover, *K. robustum*/pBBR-P1_*xfp2502*-P2_*pta2145* achieved the highest 2-KGA productivity (0.47 ± 0.034 g/L/h) and the highest l-sorbose conversion rate (Fig. 6c). While the specific production of *K. robustum*/pBBR-P1_*xfp2502*-P2_*pta2145* was 30.49 g/L/OD_600_, which displayed a slight increase compared to the wild type strain control strain (29.47 g/L/OD_600_). The results indicated that the enhancement of 2-KGA yield was mainly attributed to the increase of the final biomass. This phenomenon was in accordance with the literature reporting that the 2-KGA concentration was closely associated with the *K. vulgare* cell number [46]. Previous reports showed that the 2-KGA production of the industrial strain *K. vulgare* Hkv604 was 5.73 ± 0.04 g/L in a mono-culture system and that of the engineered strain SyBE_Kv0280002 with *hsk*-*g* was 7.17 ± 0.08 g/L [8]. For wild-type *K. vulgare* HBe602, the 2-KGA production was 5.16 ± 0.34 g/L (OD_600_ 0.701 ± 0.024) in a mono-culture system at 96 h, and production of the best performance engineered strain *K. vulgare*/pMCS2sdh was 6.0 ± 0.1 g/L (OD_600_ 0.616 ± 0.008) [23]. While in this study, the 2-KGA production and biomass was much higher than the above strains in the mono-culture system, demonstrating that *K. robustum*/pBBR-P1_*xfp2502*-P2_*pta2145* could be used as a candidate for the industrial fermentation of vitamin C.

By introducing the XFP-PTA pathway into *K. robustum* SPU_B003, the intracellular acetyl-CoA pool was increased, and the cell biomass and the conversion rate of 2-KGA were enhanced (Fig. 6d). The large pool of acetyl-CoA could contribute to the formation of higher levels of intermediates in the TCA cycle, including malate, citric acid, α-ketoglutarate, succinic acid and fumaric acid, which could facilitate the growth of *Ketogulonicigenium* sp. in the process of 2-KGA production [3, 47]. Moreover, the strong carbon flux via the TCA cycle increased the energy supply for biological activities as well as intermediates for the biosynthesis of amino acids. For example, α-ketoglutarate as an intermediate in the TCA cycle, is used to synthesize glutamate, glutamine, proline and arginine. Subsequently, glutamine is used as a major source of cellular nitrogen participates in the de novo biosynthesis of nucleotides and other nonessential amino acids. Furthermore, oxaloacetate is an important precursor for the biosynthesis of threonine, lysine and aspartate. Additionally, glycine, serine, proline, threonine and isoleucine were the key amino acids affecting cell growth and 2-KGA production [7, 8, 48]. Additionally, acetyl-CoA as a carbon-source rheostat, initiates the cellular growth by promoting the acetylation of histones at the growth genes [9]. So, presumably, the high concentration of acetyl-CoA can up-regulate the TCA cycle, consequently stimulate the relevant amino acid biosynthesis, and promote the acetylation of histones at growth genes, then facilitate cell growth and 2-KGA production.

Although the XFP-PTA pathway can theoretically achieve a 100% carbon yield to the desirable acetyl-CoA, this occurs at the expense of reducing equivalents in PPP and ATP in the EMP pathway. The abundance of NAD and NADP biosynthetic genes probably contributed to overcome the insufficiency of reducing power. In addition, the up-regulated TCA cycle could compensate for the consumption of ATP during biological activity.

### Effect of the established XFP-PTA metabolic pathway on the related gene transcription

**Fig. 7:**
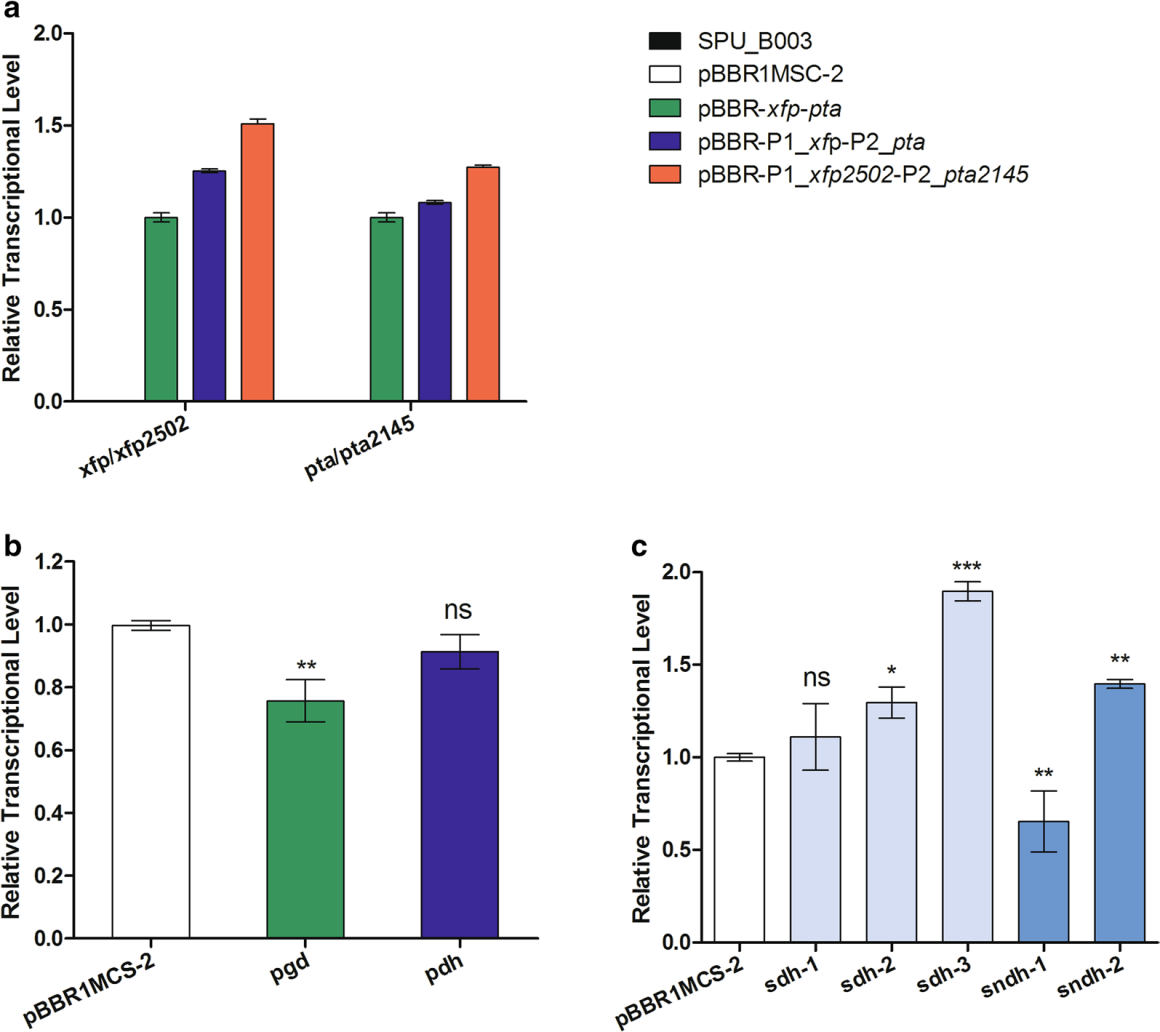
Transcriptional level of relevant genes in *K. robustum*/pBBR-P1_*xfp2502*-P2_*pta2145*. **a** Reverse transcription PCR (RT-PCR) analysis of the transcriptional information of *xfp/xfp2502* and *pta*/*pta2145*. **b** RT-qPCR analysis of the relative transcriptional level of *pgd* and *pdh* in fermentation system. **c** RT-qPCR analysis of the relative transcriptional level of *sdh* and *sndh*. Comparison was performed with control strain *K. robustum*/pBBR1MCS-2. Data represent the mean ± SD of 3 replicates. *, ** and *** Significant difference at *p* < 0.05, 0.01 and 0.001, respectively

As shown in Fig. 7a, the genes *xfp/xfp2502* and *pta/pta2145* were not transcribed in the wild type *K. robustum* SPU_B003 or control strain *K. robustum*/pBBR1MSC-2, because there were no foreign genes in the two strains. While the genes *xfp/xfp2502* and *pta/pta2145* were both transcribed in the recombinant strains, and the transcriptional levels in *K. robustum*/pBBR-P1_*xfp2502*-P2_*pta2145* and *K. robustum*/pBBR-P1_*xfp*-P2_*pta* were higher than those in *K. robustum*/pBBR-*xfp*-*pta*. This indicated that promoters P1 and P2 were stronger than the original promoters of heterologous *xfp* and *pta* in initiating gene expression in *K. robustum* SPU_B003.

To further investigate the carbon distribution in the new XFP-PTA pathway, the transcriptional levels of decarboxylation related genes were detected by RT-qPCR. As shown in Fig. 7b, the transcriptional level of *pgd* directly decreased by 24.33 ± 6.67% in *K. robustum*/pBBR-P1_*xfp2502*-P2_*pta2145* compared to the control strain, while that of the *pdh* decreased slightly (8.67 ± 5.51%). l-Sorbose can be metabolized to acetyl-CoA directly through the XFP-PTA pathway, or to pyruvate through the PPP pathway and the ED pathway, and further converted to acetyl-CoA by pyruvate dehydrogenase (PDH). The widespread source of pyruvate makes it difficult to control the transcriptional level of *pdh*, leading to a slight decrease in transcription. Whereas 6PG as the substrate of PGD is only produced in the PPP pathway. Therefore, changes in the carbon flow through the PPP pathway can easily lead to the change in the transcriptional level of *pgd*. All in all, the down-regulation of *pdh* and *pgd* means a change occurrence in carbon flux and that the CO_2_ release has been reduced to some extent.

To investigate the effects of the XFP-PTA pathway on the key genes responsible for the biosynthesis of 2-KGA, the transcriptional levels of *sdh* and *sndh* were determined by RT-qPCR. Among the five key genes, *sdh*-1 (*orf_02251*), *sdh*-2 (*orf_02271*), *sdh*-3 (*orf_p00164*) and *sndh*-2 (*orf_01127*) exhibited higher transcriptional levels than the control strain, while *sndh*-1 (*orf_00349*) decreased to some extent (Fig. 7c). One explanation for this phenomenon was that the genes of *sdh* played more important roles in the biosynthesis of 2-KGA. This corroborated a previous report showing that the 2-KGA concentration exhibited a positive linear relationship with SDH activity and quantity of *Ketogulonicigenium* sp. [46]. SDH and SNDH are the key enzymes responsible for the biosynthesis of 2-KGA, both of which are membrane-bound dehydrogenases with PQQ as cofactor. The PQQ synthesis genes in *K. vulgare* were annotated as the gene cluster *pqq*ABCDE. *pqq*A, a rate-determining step in PQQ biosynthesis, encodes a 24-amino-acid polypeptide bearing tyrosine and glutamate as building blocks [49]. Because the XFP-PTA pathway is helpful in promoting the TCA cycle and generating α-ketoglutarate, the precursor of glutamate, this route is presumably conducive to the biosynthesis of PQQ, and then affects the PQQ-regulated electron transfer in the l-sorbose oxidation process, finally affecting the transcription of *sdh* and *sndh*.

## Conclusions

In this study, a robust strain *K. robustum* SPU_B003, showing good growth characteristics and improved 2-KGA productivity, was reported. The complete genome of *K. robustum* SPU_B003 was sequenced, and the genome-based functional analysis was helpful to further understand the species-specific metabolic characteristics. Compared with *K. vulgare*, *K. robustum* SPU_B003 contained more tRNAs, rRNAs, NAD and NADP biosynthetic genes, as well as regulation- and cell signaling-related genes. At the same time, the amino acids biosynthesis pathways were more complete. In addition, two internal gene promoters were identified, and their strength was validated by detecting the initiation activity. To further enhance 2-KGA production, an innovative acetyl-CoA biosynthetic pathway was constructed by expressing phosphoketolase and phosphotransacetylase initiated by a species-specific promoter. After introducing the XFP-PTA pathway, strain *K. robustum*/pBBR-P1_*xfp2502*-P2_*pta2145* achieved the highest biomass and 2-KGA production (39.64 ± 2.84 g/L), which was significantly higher than that of *K. vulgare* Hbe602 (5.16 ± 0.34 g/L) and *K. vulgare* Hkv604 (5.73 ± 0.04 g/L) and was also increased by 22.27% compared to the control strain *K. robustum*/pBBR1MCS-2 (32.42 ± 0.96 g/L). Meanwhile, the transcriptional levels of *pgd* and *pdh* were decreased, and *sdh* and *sndh* were increased at different levels. These results suggested that the CO_2_ release was decreased and that the oxidation ability was enhanced in recombinant strain.

## Additional file

**Additional file 1: Table S1.** Codon frequency of *K. robustum* SPU_B003. **Table S2.** Optimization parameters. **Table S3.** Primers used in this study. **Table S4.** DNA sequences used in this study. **Table S5.** Growth characteristics of *K. robustum* SPU_B003. **Figure S1.** Phylogenetic analysis of *K. robustum* SPU_B003 with other species. **Figure S2.** Orthogonality test of the promoters and heterologous genes.

## Abbreviations

2-KGA: 2-keto-l-gulonic acid
*gfp*: green fluorescent protein
*xfp*: phosphoketolase
*pta*: phosphotransacetylase
*pgd*: 6-phosphogluconate dehydrogenase
*pdh*: pyruvate dehydrogenase
*sdh*: sorbose dehydrogenase
*sndh*: sorbosone dehydrogenase
TCA: tricarboxylic acid
PPP: pentose phosphate pathway
EMP: Embden–Meyerhof–Parnas
ED: Entner–Doudoroff pathway
F6P: d-fructose 6-phosphate
X5P: d-xylulose 5-phosphate
E4P: d-erythrose 4-phosphate
G3P: d-glyceraldehyde 3-phosphate
G6P: d-glucose 6-phosphate
Ru5P: d-ribulose 5-phosphate
6PG: 6-phosphogluconate
R5P: d-ribose 5-phosphate
PQQ: pyrroloquinoline quinone
