# Supplementary material for: Establishing an innovative carbohydrate metabolic pathway for efficient production of 2-keto-l-gulonic acid in Ketogulonicigenium robustum initiated by intronic promoters
Source: Microb Cell Fact. 2018 May 19;17:81. doi: 10.1186/s12934-018-0932-9 (PMC5960096; doi:10.1186/s12934-018-0932-9)
Supplement: Supplementary file 1 — Additional file 1: Table S1. Codon frequency of K. robustum SPU_B003. Table S2. Optimization parameters. Table S3. Primers used in this study. Table S4. DNA sequences used in this study. Table S5. Growth characteristics of K. robustum SPU_B003. Figure S1. Phylogenetic analysis of K. robustum SPU_B003 with other species. Figure S2. Orthogonality test of the promoters and heterologous genes. [file 12934_2018_932_MOESM1_ESM.docx]

**Additional materials**

**Establishing an innovative carbohydrate metabolic pathway for efficient production of 2-keto-L-gulonic acid in *Ketogulonicigenium robustum* initiated by intronic promoters**

Cai-Yun Wang ^a^, Ye Li ^a, b^, Zi-Wei Gao ^c^, Li-Cheng Liu ^a^, Meng-Yue Zhang ^a^, Tian-Yuan Zhang ^a^, Chun-Fu Wu ^a^, Yi-Xuan Zhang ^a,^ *

^a^ School of Life Science and Biopharmaceutics, Shenyang Pharmaceutical University, Shenyang, 110016, People's Republic of China

^b^ Northeast Pharmaceutical Group Co., Ltd, Shenyang, 110026, People's Republic of China

^c^ Department of Biotechnology, School of Engineering, Nagoya University, Furo-cho, Chikusa-ku, Nagoya 464-8603, Japan

* Corresponding Author at: School of Life Science and Biopharmaceutics, Shenyang Pharmaceutical University, 103 Wenhua Road, Shenyang, Liaoning, 110016, China.

Tel: +86-024-23986576. Fax: +86-024-23986576. E-mail: [zhangyxzsh@163.com](mailto:zhangyxzsh@163.com)

**Table S1** Codon Frequency of *K. robustum* SPU_B003

| Amino acid | codon | Frequency % |
| --- | --- | --- |
| D | GAT/GAC | 39/61 |
| E | GAG/GAA | 53/47 |
| F | TTC/TTT | 64/36 |
| G | GGA/GGC/GGG/GGT | 1/74/3/22 |
| A | GCT/GCG/GCC/GCA | 14/33/40/14 |
| C | TGT/TGC | 21/79 |
| L | TTA/TTG/CTG/CTA/CTC/CTT | 1/9/69/1/7/13 |
| M | ATG | 100 |
| N | AAT/AAC | 16/84 |
| H | CAC/CAT | 69/31 |
| I | ATT/ATC/ATA | 12/87/1 |
| K | AAG/AAA | 44/56 |
| T | ACT/ACC/ACG/ACA | 9/66/24/1 |
| W | TGG | 100 |
| V | GTC/ GTA/ GTG/ GTT | 47/2/31/19 |
| Q | CAG/ CAA | 47/53 |
| P | CCA/ CCC/ CCG/ CCT | 1/41/49/8 |
| S | TCG/TCA/TCC/AGT/AGC/TCT | 57/3/6/1/30/3 |
| R | CGA/CGC/CGT/CGG/AGG/AGA | 1/63/31/3/1/1 |
| Y | TAT/ TAC | 51/49 |
| * | TAA/TAG/TGA | 71/0/29 |

* Termination codon

**Table S2** Optimization parameters

| **Optimization parameters** |  |
| --- | --- |
| Codon usage bias | Internal chi sites and ribosomal binding sites |
| GC content | RNA instability motif (ARE) |
| mRNA secondary structure | Repeat sequences (direct repeat, reverse repeat, Dyad repeat) |
| Kozak sequence | Restriction sites that may interfere with cloning |
| Shine-Dalgarno sequence | Stop codon |

**Table S3** Primers used in this study

| **Names** | **Sequence (5’ to 3’)** | **Restriction sites** |
| --- | --- | --- |
| P1F(*gfp*) | CTGCCGGAATTCTGGGGTAAAATAATACCGTTTTTGTAACGCAC | *Eco*R I |
| P1R(*gfp*) | TGAAAAGTTCTTCTCCTTTACGCATTGTCTTTTCCAGTATACGCGGACAC | — |
| P2F(*gfp*) | CTGCCGGAATTCTTCGGTGTCAGAAGCCCTACTG | *Eco*R I |
| P2R(*gfp*) | TGAAAAGTCTTCTCCTTTACGCATGTGCGTCGTCACCTTTCGATC | — |
| P*tufB*F | CTGCCGGAATTCCAGGAGCGCATTGTTGAGCACAATG | *Eco*R I |
| P*tufB*R | GTGAAAAGTTCTTCTCCTTTACGCATCGATTGTCCCTCTAAGACACGG | — |
| *gfp*F | ATGCGTAAAGGAGAAGAACTTTTCAC | — |
| *gfp*R | CGACGCGGATCCAAAAAAAACCCCGCCCTGTCAGGGGCGGGGTTTTTTTTTTATTTGTATAGTTCATCCATGC | *Bam*H I |
| *xfp*F | CCGGAATTCTTACGCTCCCCTTATATATAGGTTCATTACTCG | *Eco*R I |
| *xfp*R | CCCAAGCTTGGCTAAGCGATGAGACTATGCTCA | *Hin*d III |
| *pta*F | GGACTAGTGCTTCACCTCAACTTCACATATAAAGATTC | *Spe* I |
| *pta*R | CCGGAATTCATGACGAGATTACTGCTGCTG | *EcoR* I |
| P1F(*xfp*) | AAGCTTTGGGGTAAAATAATACCGTTTTTGTAACGCAC | *Hin*d III |
| P1R(*xfp*) | TGTCTTTTCCAGTATACGCGGACAC | — |
| *xfp*F(P1) | GTGTCCGCGTATACTGGAAAAGACAATGTGCAGGAGTACAGGAGCAC | — |
| *xfp*R(P1) | GAATTCAAAAAAAACCCCGCCCTGTCAGGGGCGGGGTTTTTTTTTCACTCGTTGTCGCCGGC | *Eco*R I |
| P2F(*pta*) | ACTAGTTTCGGTGTCAGAAGCCCTACTG | *Spe* I |
| P2R(*pta*) | GTGCGTCGTCACCTTTCGATC | — |
| *pta*F(P2) | GATCGAAAGGTGACGACGCACGTGTCCCGTATTATTATGCTGATCCC | — |
| *pta*R(P2) | TCTAGAAAAAGGCCATCCGTCAGGATGGCCTTCTTTACTGCTGCTGTGCAGACTGAATC | *Xba* I |
| P1F(*xfp2502*) | GTCGACTGGGGTAAAATAATACCGTTTTTGTAACGCAC | *Sal* I |
| P1R(*xfp2502*) | CACCGGTCGAACGGCACATTGTCTTTTCCAGTATACGCGGACAC | — |
| *xfp2502*F | ATGTGCCGTTCGACCGGTG | — |
| *xfp2502*R | AAGCTTAAAAAAAACCCCGCCCTGTCAGGGGCGGGGTTTTTTTTTTACTCGTTGTCGCCGGCCG | *Hin*d III |
| P2F(*pta2145*) | CGGGATCCTTCGGTGTCAGAAGCCCTACTG | *Bam*H I |
| P2R(*pta2145*) | GTGCGTCGTCACCTTTCGATC | — |
| *pta2145*F | GATCGAAAGGTGACGACGCACGTCTCGCGTATCATCATGCTGATC | — |
| *pta2145*R | GCTCTAGAAAAAGGCCATCCGTCAGGATGGCCTTCTTTACTGCTGTTGGGCCGATTG | *Xba* I |
| *pdh*F | ACTTCTTCGCCAGTGATG | — |
| *pdh*R | AACCAAGGTCAGGTGTAC | — |
| *pgd*F | ATCTCGACAAGGTAGGAC | — |
| *pgd*R | CACTTCGTTAAGACCATCC | — |
| 16sF | ACAATGGTAGTGACAATGG | — |
| 16sR | GCGATTACTAGCGATTCC | — |
| *sdh*1F | GACATCATCCAAGCGATC | — |
| *sdh*1R | CCACGAGACGAAGTAAAC | — |
| *sdh*2F | CGACATCATTCAAGCGATT | — |
| *sdh*2R | AGACGAAGTAGACATTGGT | — |
| *sdh*3F | GCACATTCGAGATGATGG | — |
| *sdh*3R | CAGGTTCTGGTAGTTGGT | — |
| *sndh*1F | AATTGGATATTGCCAGCG | — |
| *sndh*1R | GTTCACGACTGTCCACAG | — |
| *sndh*2F | ATTGCGGATATAGGTGCT | — |
| *sndh*2F | CGGATCTGATGGAAGACA | — |
| *xfp*F2 | GGACAACGGATACCTCTC | — |
| *xfp*R2 | CTTGTTGGTGGACTTGAAG | — |
| *pta*F2 | AATCTCTGACTGCCACTT | — |
| *pta*R2 | AAGAACACGGAAGATACCA | — |
| *xfp2502*F2 | CGTTGATCTTATCCGCATAT | — |
| *xfp2502*R2 | AAGACCATAACGGCTTCT | — |
| *pta2145*F2 | GCCATAGACATAGACTTGTT | — |
| *pta2145*R2 | CATGAGCGTATCGAGAAG | — |

**Table S4** DNA sequences used in this study

| **Name** | **Sequence (5’ to 3’)** |
| --- | --- |
| 16s  rRNA  (1451 bp) | AGAGTTTGATCCTGGCTCAGAACGAACGCTGGCGGCAGGCCTAACACATGCAAGTCGAGCGGGACCTTCGGGTCTAGCGGCGGACGGGTTAGTAACACGTGGGAACGTACCTCTCTCTACGGAATAGCCTCGGGAAACTGAGCGTAATACCGTATACGCCCTTTGGGGGAAAGATTTATCGGAGAGAGATCGGCCCGCGTCTGATTAGATAGTTGGTGGGGTAATGGCCTACCAAGTCTACGATCAGTAGCTGGTTTGAGAGGATGATCAGCAACACTGGGACTGAGACACGGCCCAGACTCCTACGGGAGGCAGCAGTGGGGAATCTTAGACAATGGGCGCAAGCCTGATCTAGCCATGCCGCGTGAGTGAAGAAGGCCTTAGGGTCGTAAAGCTCTTTCGCTGGGGAAGATAATGACTGTACCCAGTAAAGAAACCCCGGCTAACTCCGTGCCAGCAGCCGCGGTAATACGGAGGGGGTTAGCGTTGTTCGGAATTACTGGGCGTAAAGCGCGCGTAGGCGGATTAGAAAGTTAGGGGTGAAATCCCAGGGCTCAACCCTGGAACTGCCTCTAAAACTCCTAGTCTTGAGGTCGAGAGAGGTGAGTGGAATTCCGAGTGTAGAGGTGAAATTCGTAGATATTCGGAGGAACACCAGTGGCGAAGGCGGCTCACTGGCTCGATACTGACGCTGAGGTGCGAAAGCGTGGGGAGCAAACAGGATTAGATACCCTGGTAGTCCACGCCGTAAACGATGAATGCCAGTCGTCAGGTTGCTTGCAACTTGGTGACACACCTAACGGATTAAGCATTCCGCCTGGGGAGTACGGTCGCAAGATTAAAACTCAAAGGAATTGACGGGGGCCCGCACAAGCGGTGGAGCATGTGGTTTAATTCGAAGCAACGCGCAGAACCTTACCAACCCTTGACATTACAGGACCGGCCTAGAGATAGGTCTTTCACTTCGGTGACCTGTGGACAGGTGCTGCATGGCTGTCGTCAGCTCGTGTCGTGAGATGTTCGGTTAAGTCCGGCAACGAGCGCAACCCACGTCTTTAGTTGCCAGCATTCAGTTGGGCACTCTAAAGAAACTGCCGGTGATAAGCCGGAGGAAGGTGTGGATGACGTCAAGTCCTCATGGCCCTTACGGGTTGGGCTACACACGTGCTACAATGGTAGTGACAATGGGTTAATCCCAAAAAGCTATCTCAGTTCGGATTGGGGTCTGCAACTCGACCCCATGAAGTCGGAATCGCTAGTAATCGCGTAACAGCATGACGCGGTGAATACGTTCCCGGGCCTTGTACACACCGCCCGTCACACCATGGGAGTTGGGTCTACCCGAAGGCGGTGCGCTAACCAGCAATGGAAGCAGCCGACCACGGTAGGCTCAGCGACTGGGGTGAAGTCGTAACAAGGTAGCCGTAGGGGAACCTGCGGCTGGATCACCT |
| Promoter  P1  (147 bp) | TGGGGTAAAATAATACCGTTTTTGTAACGCACTGTTTATAAACGGTTTTATCAGGTGTGGGTGGCTTGACTGGCGCGTTTAACCGCCTATAAGCCACCCATCCCAATTAGGGGCACCCACGGGTGTCCGCGTATACTGGAAAAGACA |
| Promoter  P2  (250 bp) | TTCGGTGTCAGAAGCCCTACTGGCTTCTTGAAAATCAGGCTGGGCCCGCCACTGTTGCGGGCCCAGCTTGACCTGTCTTGGAGGGTGGTTCTACCATTGCGGCATGAACCACCACCCTGGGCAGGTTCCATAGGTCGGGAGGCAACCCTTGGGACGGTTGCCGTGAATAGACCTGAACCCCCATTTCTCCATGGGCGCGCGCCGATGCCCGACGGCACGCCTCCCCAGAGATCGAAAGGTGACGACGCAC |
| Gene *xfp* and its original promoter  (2979 bp) | TTACGCTCCCCTTATATATAGGTTCATTACTCGGGCATTACAACACATCTATTGTGCGCAGGCAAGTAGACATTGAAATTCGCACATGCCACGGGTCGCATCGGTGTCGTCTTAAGCGCTCAAGCACTGCAATCACAAGGATTTTCCAGATTGTTCGCTTGTTTTCAACACGCCGCGCAATATCCTCACAAACCGCACGCGACAACGACGGCGAAAACGCTTGCATTCGTTGGTATTTCAACGTTTCTCGCCTTTATTCACTGATTTTCCATTTTCACAAATCGCCCGAGCAATCTCCCAAATTCGCAAATTATGCGCACAGATTCGCTCACACTGTTTCAAAAACTGCAAAAAGGTCAGCGTATTCGCGTAACATAATCAGCGATCGGGCACGGAGACCGGCCTGCAGGACAGCGCCGAAGCCCGTGCCCAACGGAATAAACAAATCGCACATTTATGTGCAGGAGTACAGGAGCACACATGACTAATCCTGTTATTGGTACCCCATGGCAGAAGCTGGATCGTCCGGTTTCCGAAGAGGCCATCGAAGGCATGGACAAGTACTGGCGCGTCGCCAACTACATGTCTATCGGCCAGATCTACCTGCGTAGCAACCCGCTGATGAAGGAGCCCTTCACCCGCGATGACGTGAAGCACCGTCTGGTCGGCCACTGGGGCACCACCCCGGGCCTGAACTTCCTTCTCGCCCACATCAACCGCCTGATCGCCGATCACCAGCAGAACACCGTGTTCATCATGGGTCCTGGCCACGGCGGCCCTGCAGGTACCGCTCAGTCCTACATCGACGGCACCTACACCGAGTACTACCCGAACATCACCAAGGACGAAGCTGGCCTGCAGAAGTTCTTCCGCCAGTTCTCCTACCCGGGTGGCATTCCTTCCCACTTCGCTCCGGAGACGCCGGGCTCCATCCACGAAGGCGGCGAGCTGGGCTACGCCCTGTCGCACGCCTACGGCGCGATCATGGACAACCCGAGCCTCTTCGTCCCGTGCATCATCGGTGACGGCGAAGCCGAGACCGGCCCTCTGGCCACCGGCTGGCAGTCCAACAAGCTCGTCAACCCGCGCACCGACGGCATCGTCCTGCCGATCCTGCACCTCAACGGCTACAAGATCGCCAACCCGACGATCCTCGCCCGCATCTCCGACGAGGAGCTGCACGACTTCTTCCGCGGCATGGGTTACCACCCGTACGAGTTCGTCGCCGGCTTCGACAACGAGGATCACCTGTCGATCCACCGTCGCTTCGCCGAGCTCTTCGAGACCATCTTCGACGAGATCTGCGATATCAAGGCTGCGGCTCAGACCGACGACATGACCCGTCCGTTCTACCCGATGCTCATCTTCCGCACCCCGAAGGGCTGGACCTGCCCGAAGTTCATCGACGGCAAGAAGACCGAAGGCTCCTGGCGTGCACACCAGGTCCCGCTGGCTTCCGCCCGCGACACCGAGGCCCACTTCGAAGTCCTCAAGGGCTGGATGGAATCCTACAAGCCGGAGGAGCTCTTCAACGCCGACGGCTCCATCAAGGAGGACGTCACCGCATTCATGCCTAAGGGCGAACTGCGCATCGGCGCCAACCCGAATGCCAACGGCGGCCGCATCCGCGAGGATCTGAAGCTCCCTGAACTCGATCAGTACGAGATCACCGGCGTCAAGGAATACGGCCACGGTTGGGGCCAGGTCGAGGCTCCGCGTTCCCTCGGCGCGTACTGCCGCGACATCATCAAGAACAACCCGGATTCGTTCCGCGTCTTCGGACCTGACGAGACCGCGTCCAACCGTCTGAACGCGACCTACGAGGTCACCAAGAAGCAGTGGGACAACGGATACCTCTCGGCTCTCGTCGACGAGAACATGGCCGTCACCGGCCAGGTTGTCGAGCAGCTCTCCGAGCATCAGTGCGAAGGCTTCCTCGAGGCCTACCTGCTCACCGGCCGTCACGGCATCTGGAGCTCCTACGAGTCCTTCGTGCACGTGATCGACTCCATGCTGAACCAGCATGCGAAGTGGCTCGAGGCCACCGTCCGCGAGATCCCGTGGCGTAAGCCGATCTCCTCGGTGAACCTCCTGGTCTCCTCGCACGTGTGGCGTCAGGATCACAACGGCTTCTCGCACCAGGATCCGGGTGTGACCTCCGTCCTGCTGAACAAGACGTTCAACAACGACCACGTGACGAACATCTACTTCGCGACCGATGCCAACATGCTGCTGGCCATCGCCGAGAAGTGCTTCAAGTCCACCAACAAGATCAACGCAATCTTCGCCGGCAAGCAGCCGGCCGCGACGTGGATCACCCTCGACGAGGCACGCGCCGAGCTCGAGGCTGGTGCCGCCGAGTGGAAGTGGGCTTCCAACGCCAAGAGCAACGACGAGGTCCAGGTTGTCCTCGCCGCCGCCGGCGACGTCCCGACCCAGGAGATCATGGCCGCTTCCGATGCCCTCAACAAGATGGGCATCAAGTTCAAGGTCGTCAACGTCGTGGACCTCATCAAGCTGCAGTCCTCGAAGGAGAACGACGAGGCCATGTCTGACGAGGACTTCGCCGACCTGTTCACCGCGGACAAGCCGGTCCTCTTCGCCTACCACTCCTATGCCCAGGACGTTCGTGGCCTCATCTACGACCGCCCGAACCACGACAACTTCACCGTTGTCGGATACAAGGAGCAGGGCTCCACGACGACGCCGTTCGACATGGTGCGTGTCAACGACATGGATCGCTACGCCCTTCAGGCCAAGGCCCTCGAGCTCATCGACGCCGACAAGTATGCCGACAAGATCAACGAGCTCAACGAGTTCCGCAAGACCGCGTTCCAGTTCGCCGTCGACAATGGCTATGACATTCCTGAGTTCACCGATTGGGTGTACCCGGATGTCAAGGTCGACGAGACCTCCATGCTCTCCGCCACCGCCGCGACCGCCGGCGACAACGAGTGAGCATAGTCTCATCGCTTAGCC |
| Gene *pta* and its original promoter (2492 bp) | GCTTCACCTCAACTTCACATATAAAGATTCAAAAATTTGTGCAAATTCACAACTCAGCGGGACAACGTTCAAAACATTTTGTCTTCCATACCCACTATCAGGTATCCTTTAGCAGCCTGAAGGCCTAAGTAGTACATATTCATTGAGTCGTCAAATTCATATACATTATGCCATTGGCTGAAAATTACGCAAAATGGCATAGACTCAAGATATTTCTTCCATCATGCAAAAAAAAATTTGCAGTGCATGATGTTAATCATAAATGTCGGTGTCATCATGCGCTACGCTCTATGGCTCCCTGACGTTTTTTTAGCCACGTATCAATTATAGGTACTTCCGTGTCCCGTATTATTATGCTGATCCCTACCGGAACCAGCGTCGGTCTGACCAGCGTCAGCCTTGGCGTGATCCGTGCAATGGAACGCAAAGGCGTTCGTCTGAGCGTTTTCAAACCTATCGCTCAGCCGCGTACCGGTGGCGATGCGCCCGATCAGACTACGACTATCGTGCGTGCGAACTCTTCCACCACGACGGCCGCTGAACCGCTGAAAATGAGCTACGTTGAAGGTCTGCTTTCCAGCAATCAGAAAGATGTGCTGATGGAAGAGATCGTCGCAAACTACCACGCTAACACCAAAGACGCTGAAGTCGTTCTGGTTGAAGGTCTGGTCCCGACACGTAAGCACCAGTTTGCCCAGTCTCTGAACTACGAAATCGCTAAAACGCTGAATGCGGAAATCGTCTTCGTTATGTCTCAGGGCACTGACACCCCGGAACAGCTGAAAGAGCGTATCGAACTGACCCGCAACAGCTTCGGCGGTGCCAAAAACACCAACATCACCGGCGTTATCGTTAACAAACTGAACGCACCGGTTGATGAACAGGGTCGTACTCGCCCGGATCTGTCCGAGATTTTCGACGACTCTTCCAAAGCTAAAGTAAACAATGTTGATCCGGCGAAGCTGCAAGAATCCAGCCCGCTGCCGGTTCTCGGCGCTGTGCCGTGGAGCTTTGACCTGATCGCGACTCGTGCGATCGATATGGCTCGCCACCTGAATGCGACCATCATCAACGAAGGCGACATCAATACTCGCCGCGTTAAATCCGTCACTTTCTGCGCACGCAGCATTCCGCACATGCTGGAGCACTTCCGTGCCGGTTCTCTGCTGGTGACTTCCGCAGACCGTCCTGACGTGCTGGTGGCCGCTTGCCTGGCAGCCATGAACGGCGTAGAAATCGGTGCCCTGCTGCTGACTGGCGGCTACGAAATGGACGCGCGCATTTCTAAACTGTGCGAACGTGCTTTCGCTACCGGCCTGCCGGTATTTATGGTGAACACCAACACCTGGCAGACCTCTCTGAGCCTGCAGAGCTTCAACCTGGAAGTTCCGGTTGACGATCACGAACGTATCGAGAAAGTTCAGGAATACGTTGCTAACTACATCAACGCTGACTGGATCGAATCTCTGACTGCCACTTCTGAGCGCAGCCGTCGTCTGTCTCCGCCTGCGTTCCGTTATCAGCTGACTGAACTTGCGCGCAAAGCGGGCAAACGTATCGTACTGCCGGAAGGTGACGAACCGCGTACCGTTAAAGCAGCCGCTATCTGTGCTGAACGTGGTATCGCAACTTGCGTACTGCTGGGTAATCCGGCAGAGATCAACCGTGTTGCAGCGTCTCAGGGTGTAGAACTGGGTGCAGGGATTGAAATCGTTGATCCAGAAGTGGTTCGCGAAAGCTATGTTGGTCGTCTGGTCGAACTGCGTAAGAACAAAGGCATGACCGAAACCGTTGCCCGCGAACAGCTGGAAGACAACGTGGTGCTCGGTACGCTGATGCTGGAACAGGATGAAGTTGATGGTCTGGTTTCCGGTGCTGTTCACACTACCGCAAACACCATCCGTCCGCCGCTGCAGCTGATCAAAACTGCACCGGGCAGCTCCCTGGTATCTTCCGTGTTCTTCATGCTGCTGCCGGAACAGGTTTACGTTTACGGTGACTGTGCGATCAACCCGGATCCGACCGCTGAACAGCTGGCAGAAATCGCGATTCAGTCCGCTGATTCCGCTGCGGCCTTCGGTATCGAACCGCGCGTTGCTATGCTCTCCTACTCCACCGGTACTTCTGGTGCAGGTAGCGACGTAGAAAAAGTTCGCGAAGCAACTCGTCTGGCGCAGGAAAAACGTCCTGACCTGATGATCGACGGTCCGCTGCAGTACGACGCTGCGGTAATGGCTGACGTTGCGAAATCCAAAGCGCCGAACTCTCCGGTTGCAGGTCGCGCTACCGTGTTCATCTTCCCGGATCTGAACACCGGTAACACCACCTACAAAGCGGTACAGCGTTCTGCCGACCTGATCTCCATCGGGCCGATGCTGCAGGGTATGCGCAAGCCGGTTAACGACCTGTCCCGTGGCGCACTGGTTGACGATATCGTCTACACCATCGCGCTGACTGCGATTCAGTCTGCACAGCAGCAGTAATCTCGTCAT |
| *xfp2502* (2502 bp) | ATGTGCCGTTCGACCGGTGCCCACATGACGAACCCCGTCATCGGCACGCCCTGGCAGAAACTGGATCGTCCGGTCTCGGAAGAGGCCATCGAAGGTATGGATAAGTACTGGCGTGTCGCCAACTACATGTCGATCGGCCAGATCTACCTGCGCAGCAACCCCCTGATGAAGGAGCCGTTTACCCGCGACGATGTCAAGCACCGCCTGGTCGGTCACTGGGGCACCACGCCGGGTCTGAACTTCCTGCTGGCGCATATCAACCGTCTGATCGCCGATCATCAACAGAACACCGTCTTCATCATGGGCCCCGGTCACGGTGGTCCGGCCGGCACCGCCCAAAGCTATATCGATGGCACCTACACCGAATACTACCCGAACATCACCAAGGACGAAGCCGGTCTGCAGAAGTTCTTCCGTCAGTTCAGCTACCCCGGCGGTATCCCCAGCCACTTCGCCCCGGAGACGCCGGGCAGCATCCATGAAGGCGGCGAACTGGGCTACGCCCTGTCGCATGCCTACGGCGCCATCATGGATAACCCGAGCCTGTTTGTCCCGTGTATCATCGGTGACGGTGAAGCCGAAACCGGCCCCCTGGCCACCGGCTGGCAAAGCAACAAACTGGTCAACCCGCGTACCGATGGCATCGTCCTGCCGATCCTGCATCTGAACGGTTACAAGATCGCCAACCCGACCATCCTGGCCCGTATCAGCGATGAAGAACTGCATGACTTCTTCCGCGGCATGGGCTACCATCCGTACGAGTTCGTGGCGGGCTTCGATAACGAAGATCACCTGTCGATCCACCGTCGTTTCGCCGAACTGTTTGAAACCATCTTCGACGAAATCTGCGACATCAAAGCGGCGGCGCAGACCGACGATATGACCCGCCCGTTCTACCCCATGCTGATCTTCCGCACCCCGAAGGGCTGGACCTGCCCGAAATTCATCGACGGCAAGAAGACCGAAGGCAGCTGGCGTGCGCACCAAGTCCCGCTGGCGAGCGCCCGTGATACCGAAGCCCACTTCGAGGTCCTGAAAGGCTGGATGGAATCGTACAAACCGGAAGAGCTGTTCAACGCCGATGGCTCGATCAAGGAAGATGTCACCGCGTTCATGCCCAAAGGCGAGCTGCGCATCGGCGCCAACCCGAACGCGAACGGCGGCCGTATCCGCGAGGATCTGAAACTGCCGGAACTGGACCAATATGAAATCACCGGCGTGAAAGAATATGGCCACGGCTGGGGCCAAGTCGAAGCGCCGCGCTCGCTGGGCGCGTATTGCCGCGATATCATCAAGAACAACCCCGATTCGTTCCGCGTCTTCGGCCCGGATGAAACGGCGAGCAACCGCCTGAACGCGACCTATGAAGTCACCAAGAAACAATGGGACAACGGCTATCTGTCGGCGCTGGTGGATGAGAACATGGCGGTCACCGGCCAAGTGGTCGAACAACTGTCGGAGCACCAATGCGAGGGCTTCCTGGAGGCGTATCTGCTGACCGGCCGCCACGGCATCTGGAGCAGCTATGAGTCGTTCGTGCACGTGATCGATTCGATGCTGAACCAACACGCGAAATGGCTGGAGGCCACCGTCCGCGAGATCCCGTGGCGCAAACCCATCTCGTCGGTGAACCTGCTGGTCTCGTCGCACGTGTGGCGCCAAGACCATAACGGCTTCTCGCACCAGGACCCCGGTGTGACCTCGGTCCTGCTGAACAAGACGTTTAACAACGACCACGTGACGAACATCTATTTCGCGACCGACGCCAACATGCTGCTGGCCATCGCCGAGAAATGCTTTAAATCGACCAACAAGATCAACGCGATCTTCGCGGGCAAGCAGCCCGCCGCGACGTGGATCACCCTGGACGAGGCGCGCGCGGAGCTGGAAGCGGGTGCCGCCGAGTGGAAATGGGCGTCGAACGCCAAGAGCAACGATGAGGTGCAGGTGGTCCTGGCCGCCGCGGGCGACGTCCCCACCCAAGAGATCATGGCCGCGTCGGACGCCCTGAACAAGATGGGCATCAAATTCAAAGTCGTCAACGTCGTGGACCTGATCAAACTGCAATCGTCGAAAGAGAACGACGAGGCCATGAGCGACGAGGACTTTGCCGACCTGTTTACCGCGGACAAACCCGTCCTGTTTGCCTATCACTCGTATGCCCAGGACGTGCGCGGCCTGATCTATGACCGCCCCAACCACGACAACTTTACCGTGGTCGGCTACAAAGAGCAGGGCTCGACGACGACCCCCTTTGACATGGTGCGCGTCAACGACATGGACCGCTATGCCCTGCAGGCCAAAGCGCTGGAGCTGATCGACGCCGACAAATATGCGGATAAGATCAACGAGCTGAACGAGTTTCGCAAGACGGCGTTTCAGTTTGCGGTGGATAACGGTTATGACATCCCCGAGTTTACGGACTGGGTGTATCCCGACGTGAAAGTGGACGAGACGTCGATGCTGAGCGCGACCGCGGCGACGGCCGGCGACAACGAGTAA |
| *pta2145* (2145 bp) | GTCTCGCGTATCATCATGCTGATCCCGACGGGCACGAGCGTCGGCCTGACCTCGGTCTCGCTGGGTGTCATCCGTGCCATGGAACGTAAGGGCGTCCGTCTGAGCGTCTTCAAGCCCATCGCCCAGCCGCGTACCGGTGGCGACGCCCCCGATCAAACCACGACGATCGTGCGTGCCAACAGCTCGACCACCACGGCCGCGGAACCGCTGAAGATGTCGTACGTCGAAGGTCTGCTGTCGAGCAACCAGAAGGATGTCCTGATGGAGGAGATCGTCGCCAACTACCACGCCAACACCAAAGACGCCGAAGTCGTCCTGGTCGAGGGCCTGGTCCCCACCCGTAAACACCAATTCGCCCAATCGCTGAACTACGAGATCGCCAAGACCCTGAACGCGGAAATCGTGTTCGTCATGAGCCAGGGCACGGACACCCCGGAACAGCTGAAAGAGCGCATCGAACTGACCCGCAACAGCTTCGGCGGTGCCAAGAACACGAACATCACCGGCGTGATCGTGAACAAACTGAACGCCCCGGTGGACGAGCAGGGTCGTACGCGTCCGGATCTGTCGGAGATCTTCGACGACTCGTCGAAAGCGAAAGTCAACAACGTGGACCCGGCCAAACTGCAGGAGTCGAGCCCGCTGCCCGTGCTGGGTGCCGTGCCGTGGTCGTTCGATCTGATCGCGACCCGCGCCATCGATATGGCCCGCCATCTGAACGCCACCATCATCAACGAGGGCGACATCAACACGCGCCGCGTGAAGTCGGTGACGTTCTGCGCCCGCAGCATCCCGCACATGCTGGAGCACTTCCGTGCCGGTTCGCTGCTGGTGACGAGCGCCGACCGCCCCGACGTGCTGGTGGCGGCCTGTCTGGCCGCGATGAACGGCGTGGAGATCGGTGCCCTGCTGCTGACGGGCGGCTACGAAATGGACGCCCGCATCTCGAAACTGTGCGAGCGTGCGTTCGCGACCGGCCTGCCGGTCTTCATGGTGAACACCAACACCTGGCAGACCTCGCTGAGCCTGCAGAGCTTTAACCTGGAGGTGCCCGTGGACGACCATGAGCGTATCGAGAAGGTCCAGGAGTACGTGGCGAACTACATCAACGCCGACTGGATCGAGAGCCTGACGGCGACCTCGGAGCGCAGCCGTCGTCTGTCGCCGCCCGCGTTTCGCTACCAGCTGACCGAGCTGGCGCGCAAAGCGGGCAAACGCATCGTCCTGCCGGAAGGTGACGAACCGCGCACCGTCAAAGCCGCCGCGATCTGCGCGGAACGCGGTATCGCCACCTGCGTCCTGCTGGGTAACCCGGCGGAGATCAACCGCGTGGCGGCGTCGCAAGGCGTGGAACTGGGCGCGGGCATCGAAATCGTCGATCCGGAAGTGGTCCGTGAAAGCTATGTCGGCCGCCTGGTCGAACTGCGCAAGAACAAAGGCATGACCGAAACCGTCGCCCGCGAGCAACTGGAAGATAACGTGGTGCTGGGCACGCTGATGCTGGAACAAGACGAAGTCGATGGCCTGGTGAGCGGCGCCGTGCACACCACCGCCAACACCATCCGCCCGCCGCTGCAACTGATCAAGACCGCGCCGGGCAGCTCGCTGGTCTCGTCGGTGTTTTTCATGCTGCTGCCCGAACAAGTCTATGTCTATGGCGACTGCGCGATCAACCCCGACCCCACCGCGGAGCAGCTGGCCGAAATCGCCATCCAAAGCGCGGACTCGGCGGCGGCCTTTGGCATCGAGCCCCGCGTCGCGATGCTGTCGTATTCGACCGGCACCTCGGGCGCCGGCAGCGATGTCGAGAAAGTCCGCGAAGCCACCCGCCTGGCGCAAGAGAAACGCCCGGACCTGATGATCGACGGCCCCCTGCAATATGATGCGGCGGTCATGGCGGATGTCGCGAAATCGAAGGCGCCCAACTCGCCCGTCGCCGGCCGCGCCACGGTGTTTATCTTTCCCGATCTGAACACCGGCAACACCACCTATAAGGCGGTCCAACGCTCGGCCGACCTGATCTCGATCGGCCCCATGCTGCAAGGCATGCGCAAACCCGTCAACGACCTGTCGCGCGGCGCCCTGGTGGATGATATCGTCTATACCATCGCGCTGACCGCGATCCAATCGGCCCAACAGCAGTAA |

**Table S5** Growth characteristics of *K. robustum* SPU_B003

| **Characteristics** |  |  |  |
| --- | --- | --- | --- |
| **Growth temperature (℃)** | 4-30 | **Utilization of resource** |  |
| **NaCl concentration for growth (%)** | 0-3 | Raffinose | + |
| **pH** | 6-9 | Sorbose | + |
| **Enzyme activity** |  | Fructose | + |
| Nitrate reduction | + | maltose | - |
| Urease | + | Rhamnose | - |
| Amylase | - | Arabinose | - |
| Esterase | - | Mannose | - |
| Oxidase | + | Galactose | - |
| Catalase | + | Cellose | + |
| Glucosaccharase | + | Xylose | - |
| Galactosidase | + | Xylitol | - |
|  |  | Arabinose | - |
|  |  | Disodium malate | - |
|  |  | Glucose | - |
|  |  | Mannitol | - |
|  |  | N-acetyl glucosamine | - |
|  |  | Capric acid | - |
|  |  | Adipic acid | - |
|  |  | Malic acid | - |
|  |  | Citric acid | - |
|  |  | Phenylacetic acid | - |

**+** positive reaction, - negative reaction


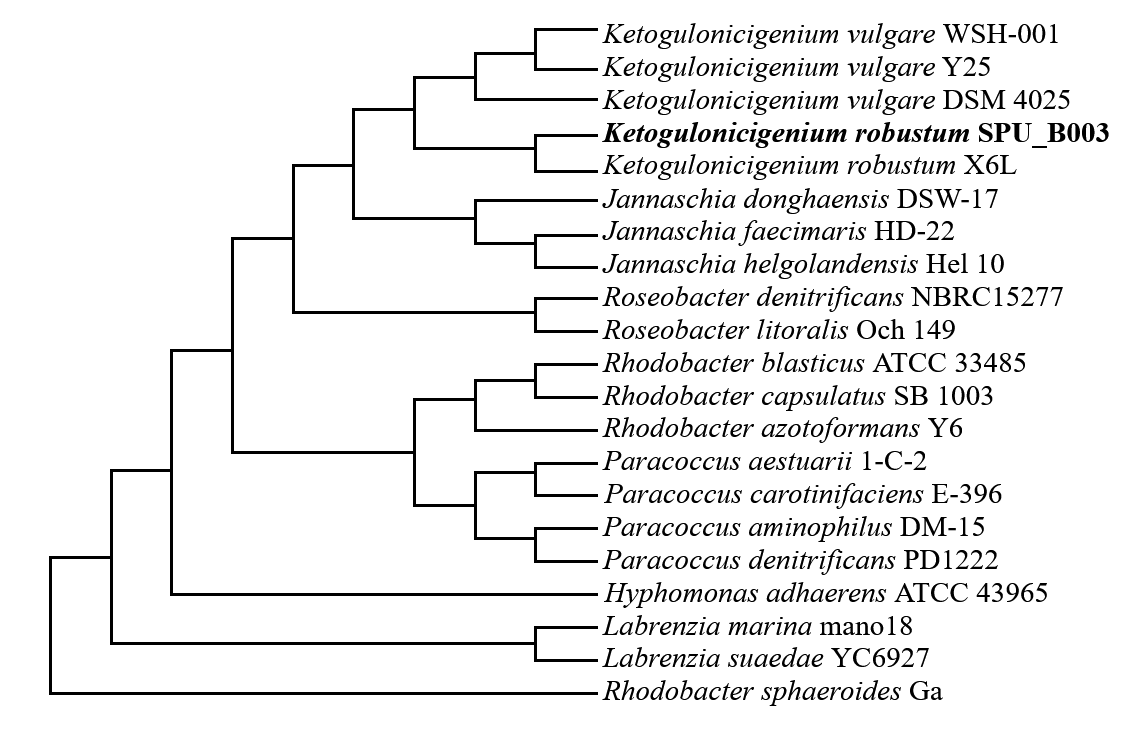


**Figure S1.** Phylogenetic analysis of *K. robustum* SPU_B003 with other species. The phylogenetic tree of phylogenetic was constructed using MEGA5 program. *Rhodobacter sphaeroides* Ga was included as an outgroup.


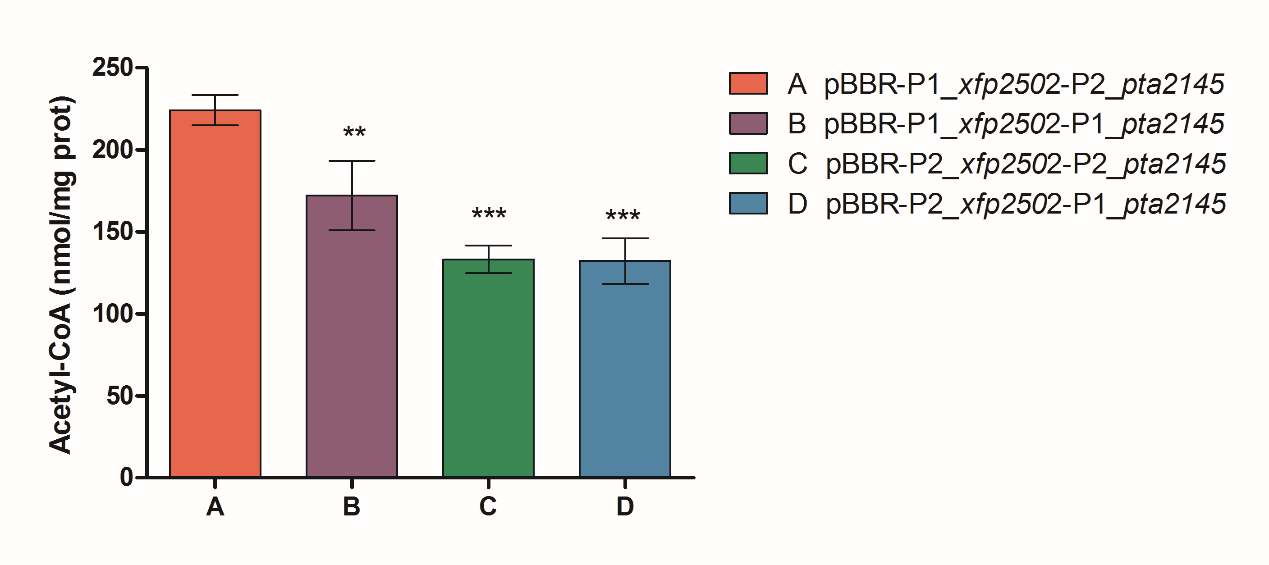


**Figure S2.** Orthogonality test of the promoters and heterologous genes. Acetyl-CoA level in different recombinant plasmids *in vivo*. Data represents the mean ± SD of 3 replicates. ** and ***, significant difference at *p* <0.01 and 0.001, respectively.
